# H2A monoubiquitination in *Arabidopsis thaliana* is generally independent of LHP1 and PRC2 activity

**DOI:** 10.1186/s13059-017-1197-z

**Published:** 2017-04-12

**Authors:** Yue Zhou, Francisco J. Romero-Campero, Ángeles Gómez-Zambrano, Franziska Turck, Myriam Calonje

**Affiliations:** 1 Max Planck Institute for Plant Breeding Research, Department of Plant Developmental Biology, Cologne, Germany; 2 Department of Computer Science and Artificial Intelligence, University of Sevilla, Seville, Spain; 3 Institute of Plant Biochemistry and Photosynthesis (IBVF-CSIC-University of Sevilla), Seville, Spain

**Keywords:** Polycomb group repression mechanism, PRC1, PRC2, H2AK121ub, H3K27me3, *Arabidopsis thaliana*

## Abstract

**Background:**

Polycomb group complexes PRC1 and PRC2 repress gene expression at the chromatin level in eukaryotes. The classic recruitment model of Polycomb group complexes in which PRC2-mediated H3K27 trimethylation recruits PRC1 for H2A monoubiquitination was recently challenged by data showing that PRC1 activity can also recruit PRC2. However, the prevalence of these two mechanisms is unknown, especially in plants as H2AK121ub marks were examined at only a handful of Polycomb group targets.

**Results:**

By using genome-wide analyses, we show that H2AK121ub marks are surprisingly widespread in *Arabidopsis thaliana*, often co-localizing with H3K27me3 but also occupying a set of transcriptionally active genes devoid of H3K27me3. Furthermore, by profiling H2AK121ub and H3K27me3 marks in *atbmi1a/b/c*, *clf/swn*, and *lhp1* mutants we found that PRC2 activity is not required for H2AK121ub marking at most genes. In contrast, loss of AtBMI1 function impacts the incorporation of H3K27me3 marks at most Polycomb group targets.

**Conclusions:**

Our findings show the relationship between H2AK121ub and H3K27me3 marks across the *A. thaliana* genome and unveil that ubiquitination by PRC1 is largely independent of PRC2 activity in plants, while the inverse is true for H3K27 trimethylation.

**Electronic supplementary material:**

The online version of this article (doi:10.1186/s13059-017-1197-z) contains supplementary material, which is available to authorized users.

## Background

Polycomb group (PcG)-mediated epigenetic marks contribute to maintain the transcriptionally repressed state of genes involved in important cellular and developmental processes in eukaryotes [1, 2]. PcG proteins are found in two major protein complexes, Polycomb repressive complex 2 (PRC2), which has histone H3 lysine 27 (H3K27) tri-methyltransferase activity [3], and PRC1, which has histone H2A E3 ubiquitin ligase activity [4] as well as other non-enzymatic functions critical for chromatin compaction [5].

Vertebrate PRC2 comprises EZH2 (or its closely related EZH1), which is the catalytic subunit, EED, SUZ12, and RBBP46 (or RBBP48) [6, 7]. Homologs of these components are also found in *Drosophila* [6, 7] and plants [8–10]. In *Arabidopsis thaliana* PRC2 encompasses the EZH2 homologs CURLY LEAF (CLF) [11], SWINGER (SWN) or MEDEA (MEA) [12, 13], the SUZ12 homologs EMBRYONIC FLOWER 2 (EMF2) [14], VERNALIZATION 2 (VRN2) or FERTILIZATION INDEPENDENT SEED 2 (FIS2) [15, 16], the EED equivalent FERTILIZATION INDEPENDENT ENDOSPERM (FIE) [17], and the RBBP46/48 homolog SUPPRESSOR OF IRA 1 (MSI1) [18]. While CLF and SWN are the catalytic subunits of the different combinational PRC2s acting during sporophyte development [12], MEA confers enzymatic activity to the complex during gametophyte and early seed formation [19, 20].

The vertebrate PRC1 E3 monoubiquitin ligase module comprises RING1B (or RING1A) and one of the six Polycomb RING finger (PCGF) proteins, while the one in *Drosophila* is constituted by dRing and Psc, Su(z)2, or L(3)73 Ah [7, 21]. The E3 monoubiquitin ligase module can associate with PHC1/2/3 and CBX2/4/6/7/8 (Ph and Pc, respectively, in *Drosophila*) to constitute canonical PRC1s or with other subunits to form variant PRC1s [6, 7]. In *A. thaliana* the module includes one of three AtBMI1s (AtBMI1A/B/C) and AtRING1A or AtRING1B [22–24]. Besides these conserved subunits, there are plant-specific proteins that participate in PcG-mediated gene repression, playing a role that is not yet well-defined [25, 26]. Such is the case of LIKE HETEROCHROMATIN PROTEIN 1 (LHP1), which has been proposed to be the functional equivalent to vertebrate CBX proteins or *Drosophila* Pc due to its ability to bind H3K27me3 [27, 28] and interact with other PRC1 components [22, 29, 30]; however, it also co-purifies with PRC2 [31, 32].

Since the identification of PcG proteins, an immense amount of biochemical work has focused on understanding the PcG repression mechanism. A major issue has been to determine the sequence of events. The recruitment of PcG complexes to specific targets in animals has been widely thought to occur in two steps: first PRC2 incorporates H3K27me3 at a specific gene, and then the PRC1 complex is recruited by its ability to bind to H3K27me3 to mediate H2A monoubiquitination [33]. This classic hierarchical model was also adopted by the plant field despite very limited supporting evidence. However, recent results indicate that PRC1 recruitment may occur via H3K27me3-dependent and -independent mechanisms [34] and, furthermore, that PRC1, in some cases, recruits PRC2 [24, 35–37]. The prevalence of these possible mechanisms is unclear, especially in plants, as H2AK121ub marks have been examined at only a handful of PcG targets and the interdependence of PRC1 and PRC2 remains an unanswered key question.

Our genome-wide chromatin data in PcG mutants in *A. thaliana* revealed that PRC2 activity and H3K27me3 marking do not act upstream of H2A monoubiquitination in the regulation of most genes, which strongly argues against the classic model for PcG mark deposition as the prevailing mechanism. Furthermore, LHP1 is fully dispensable for H2A monoubiquitination, indicating that a non-canonical PRC1 is responsible for all H2AK121 monoubiquitination in *A. thaliana* and that this complex can find target regions independently of H3K27me3. In contrast, the activity of this non-canonical PRC1 is required for H3K27me3 coverage at the majority of PcG target loci since these display reduced levels of both H2AK121ub and H3K27me3 in *atbmi1a/b/c* mutants*.*

## Results and discussion

### H2AK121ub marks are widely distributed in the *A. thaliana* genome, often co-localizing with H3K27me3

**Fig. 1:**
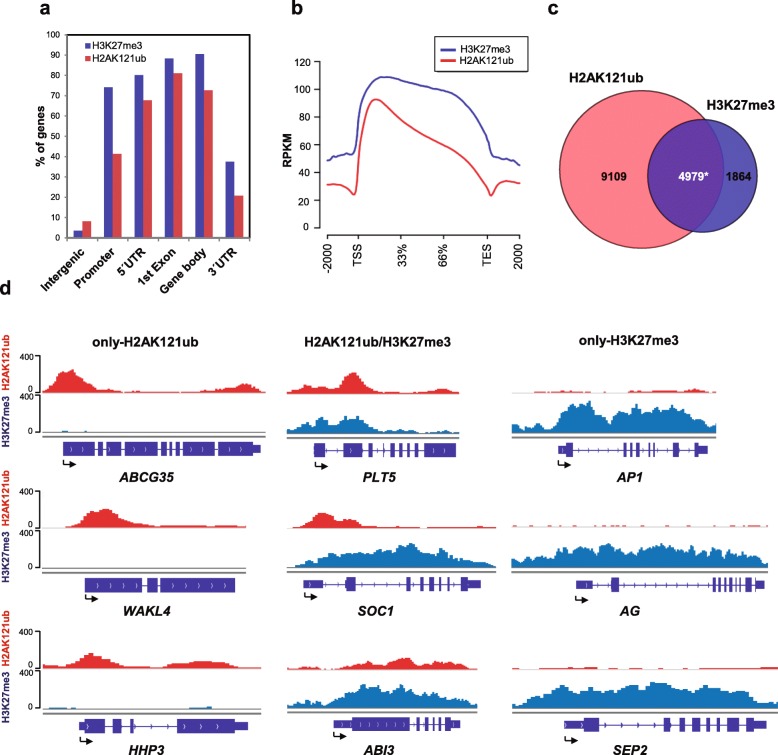
Genome-wide occupancy of H2AK121ub and H3K27me3 marks in *A. thaliana*. **a** Percentage of genes showing H2AK121ub and H3K27me3 peaks at annotated genic and intergenic regions in the *A. thaliana* genome. **b** Metagene plots of H2AK121ub and H3K27me3 coverage at target genes. *TES* transcription end site, *TSS* transcription start site. **c** Overlap between H2AK121ub- and H3K27me3-marked genes in WT at 7 DAG. *Asterisk* indicates significant overlap with *p* value <2.2 × 10^−16^ and odds ratio 1.74 according to Fisher’s exact test. **d** ChIP-seq genome browser views of H2AK121ub and H3K27me3 occupancy at selected genes. Gene structures and names are shown underneath each panel. *Arrows* indicate TSSs

To investigate the sequence of events in *A. thaliana* PcG mark deposition, we first mapped the genome-wide localization of H2AK121ub and H3K27me3 marks in wild-type Columbia-0 (Col-0, WT) seedlings 7 days after germination (DAG) by chromatin immunoprecipitation followed by sequencing (ChIP-seq). We found that H2AK121ub marks were surprisingly widespread in *A. thaliana* as 14,088 genes were associated with these, whereas 6843 were H3K27me3 marked (Additional file 1: Figure S1; Additional file 2: Dataset S1). Since the number of H2AK121ub peaks was unexpectedly high, two different peak-calling methods were employed, which generated largely identical results (Additional file 1: Figure S2). Widespread localization of H2AK118ub marks has also been recently reported in animals, where the impact of this modification at most loci is not yet understood [21]. Distribution analysis of H2AK121ub and H3K27me3 peaks across the genome and metagene analysis showed that both marks were generally targeted to gene regions (Fig. 1a, b); however, H3K27me3 peaks were significantly longer than H2AK121ub peaks (*p* value of 2.2 × 10^–16^ according to Wilcoxon test), covering on average 1.7 kb and 0.6 kb, respectively (Additional file 1: Figure S3). Using a random permutation test, we found that the overlap of H2AK121ub and H3K27me3 peaks with the promoter and different gene regions was significant (Bejamini–Hochberg corrected *p* values of the order of 10^–3^); however, while 80% of the H2AK121ub-marked genes presented a peak overlapping with their first exon (Fig. 1a), a similar percentage of H3K27me3-marked genes presented a peak overlapping with the promoter, 5′ UTR, first exon, and gene body (Fig. 1a), indicating that the majority of H3K27me3 peaks occupy genes spreading into their promoter regions whereas H2AK121ub peaks remain centered around the first exon.

We found that H2AK121ub and H3K27me3 peaks often marked the same genes (H2AK121ub/H3K27me3; 4979 genes); however, a surprisingly high number of genes were also exclusively marked with H2AK121ub (only-H2AK121ub; 9109 genes) and a lower but considerable number of genes were only marked with H3K27me3 (only-H3K27me3; 1864 genes) (Fig. 1c, d; Additional file 2: Dataset S1; Additional file 1: Figure S4). These three differently marked subsets of genes have also been recently reported in animals [21, 38]. To identify possible differences between the two subsets of H2AK121ub-marked genes, we compared H2AK121ub coverage at H2AK121ub/H3K27me3- and only-H2AK121ub-marked genes (Additional file 1: Figure S5). We found higher levels of H2AK121ub in gene bodies of H2AK121ub/H3K27me3- compared to only-H2AK121ub-marked genes (*p* value of 2.2 × 10^–16^ according to Wilcoxon test), suggesting that H3K27me3 has an effect on H2AK121ub distribution.

**Fig. 2:**
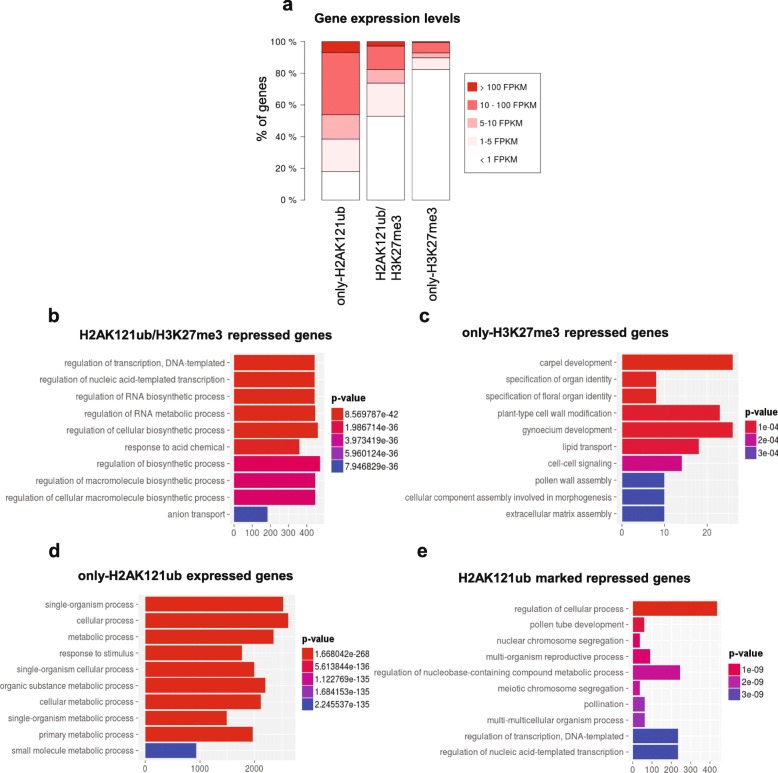
Expression levels of differentially marked genes in *A. thaliana* WT seedlings at 7 DAG. **a** Percentage of genes belonging to different expression level categories for only-H2AK121ub-, H2AK121ub/H3K27me3-, and only-H3K27me3-marked genes. Expression levels are indicated in fragments per kilobase of exon per million fragments mapped (FPKM). **b** Gene ontology (GO) enrichment analysis of H2AK121ub/H3K27me3-repressed genes (below 5 FPKM). **c** GO enrichment analysis of only-H3K27me3-repressed genes. **d** GO enrichment analysis of only-H2AK121ub-marked expressed genes (at least 5 FPKM). **e** GO enrichment analysis of onlyH2AK121ub-repressed genes. Distribution of enriched GO terms into the different “biological process” categories as defined by TAIR. *P* values are indicated by *color*, the number of genes per category is indicated on the *x-axes* for **b**–**e**

To determine the transcriptional states of these differently marked genes, we analyzed their steady-state transcript levels by RNA-seq in WT at 7 DAG (Additional file 2: Dataset S1; Additional file 1: Figure S6). Most of H2AK121ub/H3K27me3 and only-H3K27me3 genes were not expressed or displayed very low expression levels (Fig. 2a), consistent with a repressive nature of these marks. Gene Ontology (GO) analyses showed that H2AK121ub/H3K27me3-repressed genes were enriched for GO terms related to transcriptional regulation (Fig. 2b), including different families of transcription factors (Additional file 1: Figure S7), while only-H3K27me3-repressed targets showed specific enrichment for floral organ identity genes (Fig. 2c), among which the MADS box transcription factors were significantly overrepresented (Additional file 1: Figure S7). Surprisingly, 60% of only-H2AK121ub genes were non-canonical PcG targets as they were transcriptionally active and predominantly involved in metabolic processes (Fig. 2a, d). The remaining 40% were lowly or not expressed genes involved in diverse cellular processes (Fig. 2a, e). A recent report in humans presented a PRC1 variant that binds and H2A monoubiquitinates genes involved in metabolism that are devoid of H3K27me3 and have a transcriptionally active chromatin profile [38]. A repressive role of H2A monoubiquitination at these genes is possibly overridden by the presence of other chromatin modifications involved in transcription activation or PRC1 might have a role in transcriptional activation, as has been previously proposed [39]. In any case, the distribution of H2AK121ub marks in the two subsets of only-H2AK121ub-marked genes was similar (Additional file 1: Figure S5).

### Neither PRC2 activity nor LHP1 function are a major determinant for H2A monoubiquitination in *A. thaliana*

**Fig. 3:**
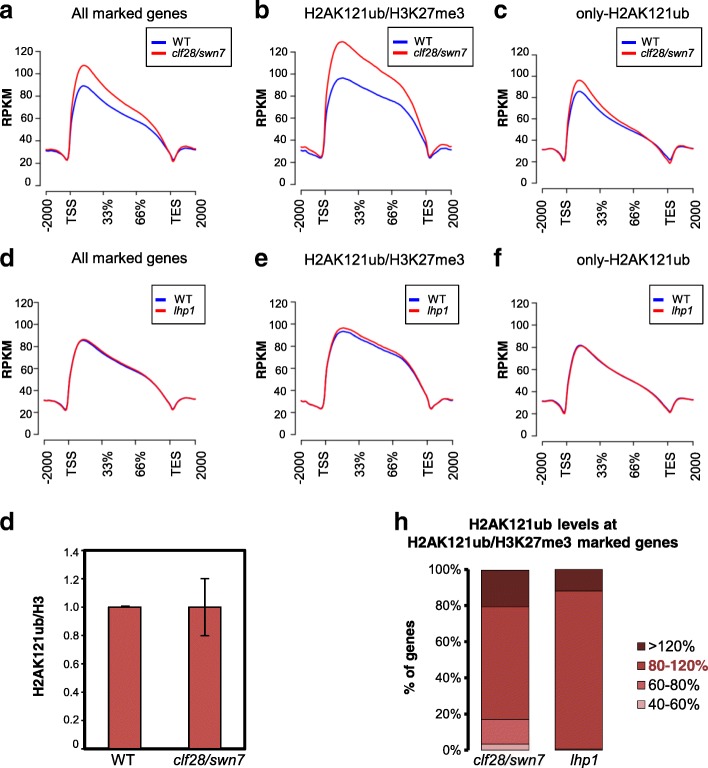
H3K27me3 marks and LHP1 are dispensable for H2AK121ub marking in *A. thaliana*. **a**–**c** Metagene plot showing H2AK121ub coverage at **a** all marked genes, **b** H2AK121ub/H2AK121ub/H3K27me3-marked genes,and **c** only-H2AK121ub-marked genes in WT and *clf28/swn7* mutants. **d**–**f** Metagene plot showing H2AK121ub coverage at **d** all marked genes, **e** H2AK121ub/H2AK121ub/H3K27me3-marked genes, and **f** only-H2AK121ub-marked genes in WT and *lhp1* mutants. **g** Western blot quantification of H2AK121ub levels normalized to H3 levels in *clf28/swn7. Error bars* represent standard deviation among at least three biological replicates (see also Additional file 1: Figure S9). **h** Levels of H2AK121ub marks at H2AK121ub/H3K27me3-marked genes in *clf28/swn7* and *lhp1* mutants compared to WT. Percentage of genes with different levels of the marks is indicated by the *shade of red* (80–120% is considered WT levels)

As the classic model of PcG repression in animals proposes that PRC2-mediated H3K27me3 recruits PRC1 that in turn monoubiquitinates H2A [33], we examined whether this model could be supported by experimental data in *A. thaliana*. We compared the genome-wide localization of H2AK121ub in *clf28/swn7* double mutants, in which H3K27me3 marks nearly disappear [40], and WT seedlings at 7 DAG (Additional file 1: Figure S8). A metagene plot of H2AK121ub coverage at target genes showed a significant increase of H2AK121ub in mutants compared to WT (Fig. 3a; *p* value of 2.2 × 10^–16^ according to Wilcoxon test). The same result was obtained when building the density heatmap of H2AK121ub marks in WT and *clf28/swn7* (Additional file 1: Figure S9). When we analyzed the coverage at H2K121ub/H3K27me3 and only-H2AK121ub genes separately (Fig. 3b, c), we found that the global change of H2AK121ub levels in *clf28/swn7* was due to a significant increase in the levels at H2AK121ub/H3K27me3-marked genes (*p* value of 2.2 × 10^–16^ according to Wilcoxon test); nevertheless, this global increase of H2AK121ub levels could not be clearly appreciated by western blot analysis (Fig. 3g; Additional file 1: Figure S9).

To further evaluate the extent of the increase in H2AK121ub at target genes, we normalized peak read coverage using robust linear regression computed over common peaks with ±20% change (mutant versus WT) in coverage measured as reads per million mapped (RPM). This constitutes a variation of the MAnorm approach [41] (“Methods”; Additional file 1: Figure S10). The normalized values were then used for quantitative comparison. We found that despite the fact that most H2K121ub/H3K27me3-marked genes showed unaltered or even increased levels in *clf28/swn7*, there was also a small percentage of genes displaying a considerable reduction (Fig. 3h; Additional file 3: Dataset S2), which was validated by ChIP-quantitative PCR (qPCR) analysis (Additional file 1: Figure S11). These data reveal that PRC2 activity is not required for establishing H2AK121ub at most target genes; however, in the absence of this activity the levels of these marks are not appropriately maintained at some genes.

Since the binding of Pc to H3K27me3 marks has been proposed to recruit PRC1 for H2A monoubiquitination [33], we compared the profile of H2AK121ub marks in *lhp1* mutants and WT (Additional file 1: Figure S8). Metagene plots of H2AK121ub coverage at all marked genes or at H2K121ub/H3K27me3- and only-H2AK121ub-marked genes separately did not show differences between *lhp1* mutants and WT (Fig. 3d–f). The same result was obtained when comparing the density heatmap in WT and *lhp1* (Additional file 1: Figure S9). In agreement with this, the levels at H2AK121ub peaks after normalization (Additional file 1: Figure S10) were similar in *lhp1* and WT (Fig. 3h; Additional file 4: Dataset S3). All together, these data indicate that LHP1 is dispensable for H2AK121ub marking in *A. thaliana*.

### The levels of H2AK121ub and H3K27me3 are significantly affected in *atbmi1a/b/c* mutants

**Fig. 4:**
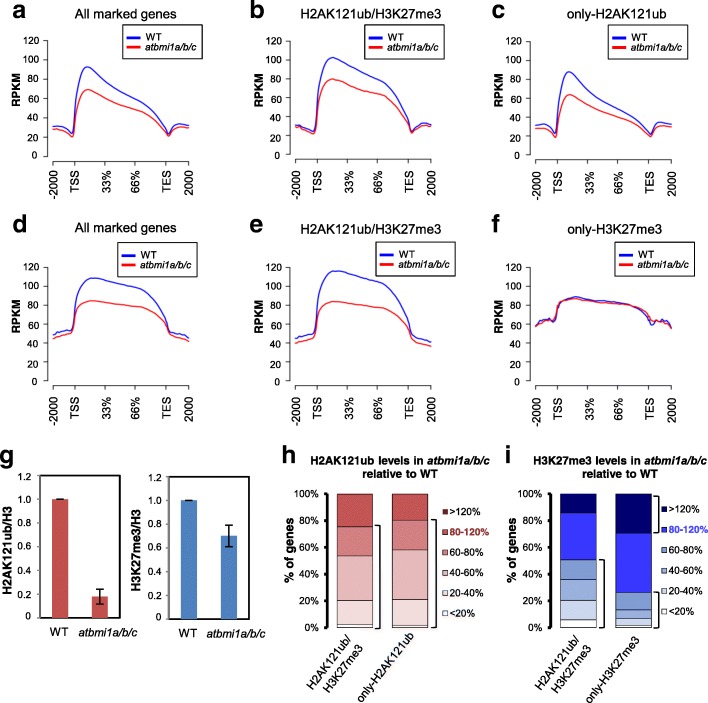
H2AK121ub and H3K27me3 marks are reduced in *atbmi1a/b/c* mutants. **a**–**c** Metagene plot showing H2AK121ub coverage at **a** all marked genes, **b** H2AK121ub/H3K27me3-marked genes, and **c** only-H2AK121ub-marked genes in WT and *atbmi1a/b/c* mutants. **d**–**f** Metagene plot showing H3K27me3 coverage at **d** all marked genes, **e** H2AK121ub/H3K27me3-marked genes, and **f** only-H3K27me3-marked genes in WT and *atbmi1a/b/c* mutants. **g** WB quantification of H2AK121ub and H3K27me3 levels normalized to H3 levels in WT and *atbmi1a/b/c. Error bars* represent standard deviation among at least three biological replicates (see also Additional file 1: Figures S13 and S18). **h**, **i** Percentage of genes retaining different levels of **h** H2AK121ub and **i** H3K27me3 marks at peaks in *atbmi1a/b/c* mutants. H2AK121ub levels are indicated by the *shade of red* and H3K27me3 levels by the *shade of blue* (80–120% is considered WT levels)

AtBMI1 proteins were shown to be involved in H2A monoubiquitination [22–24]; we therefore compared H2AK121ub profiles in WT and *atbmi1a/b/c* triple mutants at 7 DAG (Additional file 1: Figure S12). A metagene plot and heatmap of H2AK121ub coverage at target genes (Fig. 4a; Additional file 1: Figure S13) showed that H2AK121ub levels were significantly decreased in mutants compared to WT (*p* value of 2.2 × 10^–16^ according to Wilcoxon test). A global reduction of H2AK12ub marks was further supported by WB and ChIP-qPCR analyses (Fig. 4g; Additional file 1: Figures S13 and S14). Although both H2AK121ub/H3K27me3- and only-H2AK121ub-marked genes displayed significantly decreased levels (Fig. 4b, c; *p* value of 2.2 × 10^–16^ for both), note that the marks were not completely lost. In addition, the loss of H2AK121ub marks observed in metagene plots was apparently smaller than the one detected by WB analysis. This is in part explained by technical limitations as libraries prepared from *atbmi1a/b/c* mutants yielded less DNA and showed reduced mappability (Additional file 1: Table S1), which likely causes an underestimation of the loss of H2AK121ub levels at peaks after normalization (see “Methods”; Additional file 1: Figure S13). Nonetheless, the in vivo H2A E3 monoubiquitin ligase activity in animals resides in RING1A/B, while BMI1 stimulates this activity [42]; hence, a similar scenario in *A. thaliana* may lead to differential sensitivity of H2AK121ub sites to the loss of AtBMI1 function. To determine to what extent the levels of H2AK121ub were dependent on AtBMI1 activity, we quantified H2AK121ub levels at peaks in the *atbmi1a/b/c* mutant compared to WT after normalization (Fig. 4h; Additional file 5: Dataset S4; see also “Methods” and Additional file 1: Figures S15 and S16). Around 80% of H2AK121ub-marked genes showed reduced levels of H2AK121ub at their associated peaks in *atbmi1a/b/c* mutants (Fig. 4h; Additional file 1: Figure S17) to clearly varying degrees, ranging from less than 20% to 80% of WT levels (Fig. 4h). Therefore, loss of AtBMI1 function impacts the incorporation of H2AK121ub marks to different degrees depending on the gene, suggesting that the stimulating activity of AtBMI1 over AtRING1 is context-dependent.

Next, we examined whether H3K27me3 marking was affected in *atbmi1a/b/c* mutants by comparing H3K27me3 profiles in WT and mutant plants (Additional file 1: Figure S12). A metagene plot and heatmap of H3K27me3 coverage in WT and *atbmi1a/b/c* mutants (Fig. 4d; Additional file 1: Figure S18) showed a significant reduction in global H3K27me3 levels in the mutants (*p* value of 2.2 × 10^–16^ according to Wilcoxon test), which was confirmed by western blot analysis (Fig. 4g; Additional file 1: Figure S18). A global reduction in levels of H3K27me3 in *atbmi1a/b/c* was correlated with the decrease of the marks at H2AK121ub/H3K27me3 genes (Fig. 4e, f). To further investigate the impact of the loss of AtBMI1 function on H3K27me3 levels, we quantified the changes of the levels at H3K27me3 peaks in *atbmi1a/b/c* mutants (Fig. 4i; Additional file 1: Figures S19 and S20; Additional file 6: Dataset S5). Fifty percent of H2AK121ub/H3K27me3 genes displayed, to some extent, decreased levels of H3K27me3 at their associated peaks in the mutants (Fig. 4i). Furthermore, H3K27me3 peaks were severely reduced at some of these genes, indicating that loss of AtBMI1 function affects the deposition or the maintenance of H3K27me3 marks. We also found that 10% of the genes exhibited increased levels of H3K27me3 marks (Fig. 4i). Increased levels of H3K27me3 have been previously reported at some loci in *atring1a/b* and *atbmi1a/b* double mutants [43]; however, it is not known whether this is a consequence of unbalanced PcG activities or an indirect effect of the global gene misregulation experienced by these mutants [44]. Surprisingly, around 20% of only-H3K27me3 genes showed decreased or increased levels of H3K27me3 marks at peaks in *atbmi1a/b/c* mutants (Fig. 4i; Additional file 6: Dataset S5). It is possible that AtBMI1s target and affect these genes by an H2AK121ub-independent mechanism given that H2A monoubiquitination is dispensable for repression of some PRC1 targets in animals, while other BMI-mediated functions are still required [45, 46].

### Levels of H3K27me3 and H2AK121ub are correlated

**Fig. 5:**
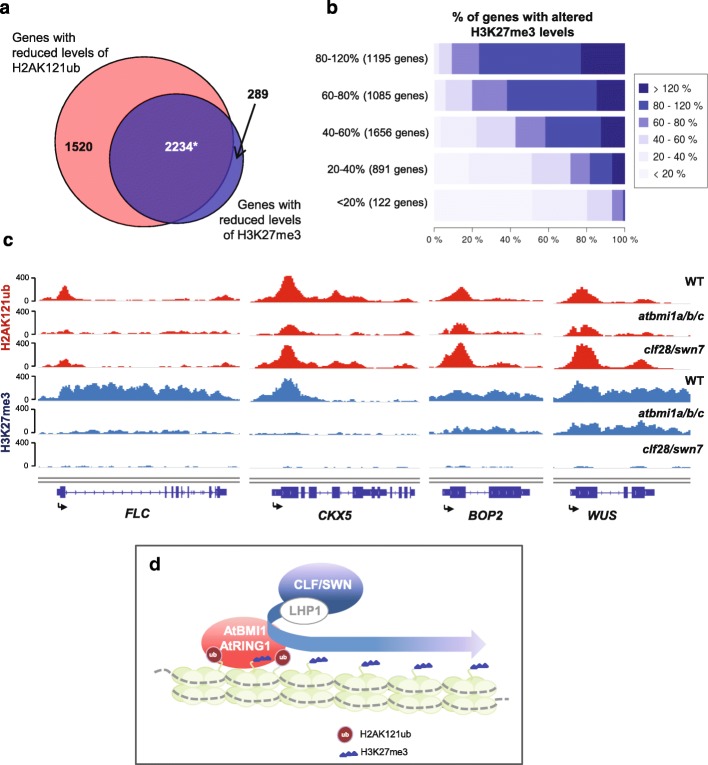
Levels of H3K27me3 and H2AK121ub marks are correlated. **a** Overlap between genes with reduced levels of H2AK121ub and H3K27me3 marks in *atbmi1a/b/c* mutants (<80% of WT levels). *Asterisk* indicates significant overlap with *p* value <2.2 × 10^−16^ and odds ratio of 7.95 according to Fisher’s exact test. **b** Correlation of H2AK121ub and H3K27me3 levels in *atbmi1a/b/c* mutants. H2AK121ub-marked genes were partitioned to consecutive categories ranked by the percentage of H2AK121ub marks at peaks in mutants compared to WT (category on the *y-axis*). The number of genes in each category is indicated. The *x-axis* indicates the percentage of genes displaying different changes in H3K27me3 marks. Categories for change in H3K27me3 levels are indicated by the *shade of blue*. **c** ChIP-seq genome browser views of H2AK121ub and H3K27me3 levels at different genes in WT, *atbmi1a/b/c*, and *clf28/swn7* mutants. Gene structures and names are shown underneath each panel. *Arrows* indicate transcription start sites. **d** Proposed model for a requirement of PRC1 activity to establish H3K27me3 marks at H2AK121ub/H3K27me3 marked genes. Although LHP1 interacts with PRC1, its function is not required for H2AK121 monoubiquitination

To ascertain if altered levels of H2AK121ub and H3K27me3 at H2AK121ub/H3K27me3 genes in *atbmi1a/b/c* mutants reflect an interdependence of these marks, we compared the number of H2AK121ub/H3K27me3 genes that displayed decreased levels of H2AK121ub with those with reduced levels of H3K27me3 in *atbmi1a/b/c*. Most of the genes showing decreased levels of H3K27me3 were included in the subset of genes with reduced H2AK121ub (Fig. 5a). To further examine how H3K27me3 levels were correlated to H2AK121ub levels, we partitioned H2AK121ub/H3K27me3 genes to consecutive categories ranked by the percentage of H2AK121ub marks at their associated peaks in mutants compared to WT. Then, we determined the fraction of genes in each category showing differential levels of H3K27me3 in mutants. Genes displaying strongly decreased levels of H2AK121ub were also severely depleted in H3K27me3 (Fig. 5b, c), whereas genes that maintained H2AK121ub levels also kept higher levels of H3K27me3 (Fig. 5b, c). Taken together, these results indicate a requirement of H2A monoubiquitination to establish and maintain appropriate H3K27me3 levels.

## Conclusions

Our findings show that LHP1 is not required for H2AK121ub marking and that PRC2 activity is dispensable to establish H2AK121ub marks at most genes, which argues against the classic hierarchical model for PcG mark deposition as the prevailing sequence of events in *A. thaliana*. Nevertheless, we found that around 20% of H2AK121ub/H3K27me3-marked genes showed to some extent decreased H2AK121ub levels in the absence of PRC2 activity. It could be possible that PRC2 activity is required to maintain appropriate H2AK121ub levels at these genes. Alternatively, loss of H2AK121ub might be a consequence of the transcriptional activation of these genes. Moreover, we found that AtBMI1 activity is required for establishing and maintaining proper H3K27me3 levels at H2AK121ub/H3K27me3 genes (Fig. 5d). According to this, H2A monoubiquitination has been shown to promote H3K27me3 [37]. However, the fact that H2AK121ub coverage is more similar to that of H3K27me3 in H3K27me3/H2AK121ub-marked genes than in only-H2AK121ub genes suggests a positive feedback loop for H2A monoubiquitination. Positive feedback loops for generating PcG-repressed chromatin has been previously proposed in animals [37]. Interestingly, loss of AtBMI1 function seems to have an effect on the levels of H3K27me3 at some only-H3K27me3-marked genes, which is a priori surprising but consistent with studies showing that PRC1 ubiquitin-independent functions are required for the repression of some targets in animals [45, 46]. On the other hand, a recent report revealed that the H2A deubiquitinases UBIQUITIN SPECIFIC PROTEASE (UBP) 12 and UBP13 are needed for H3K27me3 marking and repression of a subset of PcG targets in *A. thaliana* [47]. Similarly, *Drosophila* Calypso deubiquitinase has been proposed to remove or balance H2A monoubiquitination levels for appropriate repression [48, 49]. Therefore, it might be possible that H2AK121ub marks are initially incorporated at only-H3K27me3 genes but then removed.

## Methods

### Plant material and growth conditions

*A. thaliana* Col-0 wild type (WT), *atbmi1a/b/c* [24], *clf28/swn7* [40] and *lhp1* (also named *tfl2-2* [50]) mutants were grown under long-day conditions at 21 °C on MS agar plates containing 1.5% sucrose and 0.8% agar for 7 days.

### ChIP-seq and ChIP-qPCR

ChIP experiments were performed as previously described [51]. Chromatin was extracted from 7-day-old whole seedlings (150 seedlings). Anti-H2Aub (Cell Signaling Technology, 8240S) and anti-H3K27me3 (Diagenode, C15410069) antibodies were used for chromatin immunoprecipitation. For ChIP-seq, two immunoprecipitations from independent biological replicates were processed for next-generation sequencing library preparation. All libraries were prepared by the Ovation® Ultralow Library Systems (NuGEN) following the manufacturer’s instruction using 80% of a typical ChIP as starting material. After amplification for 16 PCR cycles, DNA of a size range between 200 and 500 bp was purified from an agarose gel. Amplification was confirmed by testing an aliquot of the library before and after amplification by qPCR. Sequencing was carried out as single-end 100-nucleotide reads on an Illumina HiSeq by the Max Planck Genome Centre in Cologne. For ChIP-qPCR, amplification was performed using Sensi FAST SYBR & Fluorescein kit (Bioline) and an iQ5 Biorad system. Samples were normalized to input DNA prepared from a reverse cross-linked aliquot of each chromatin preparation. qPCR data are shown as the means of two replicates from a representative experiment. Primers used for ChIP-qPCR are shown in Additional file 1: Table S2.

### Quality control and read mapping

Read quality of each sequenced sample was examined using the software package FASTQC (http://www.bioinformatics.babraham.ac.uk/projects/fastqc/). No quality problem was detected in the sequenced samples. The *A. thaliana* genome sequence TAIR10 in fasta format and its corresponding gene annotation in GTF format were downloaded from the data base Ensembl plants (http://plants.ensembl.org/) release 23 and used as the reference genome. Read mapping to the *A. thaliana* reference genome was carried out using the ultrafast, memory-efficient short read aligner bowtie [52]; the parameters -v 2 --best --strata -m 1 were used to allow at most two mismatches and report only the best alignment when multiple ones were found. High percentages of mapped reads were produced for each sample and no problems were detected during the mapping process (Additional file 1: Table S1). The bowtie output was stored in SAM format. SAM to BAM format conversion, sorting, and indexing were performed with the software package SAMtools [53].

### Peak calling and annotation

The software package MACS2 [54] was used for the identification of read-enriched regions or peaks. The software tool SICER [55] was used to check the robustness of our results for H2AK121ub. Indeed, 93.7% of the peaks detected by MACS2 were also detected by SICER (Additional file 1: Figure S2). A common input library and default parameters were used for all samples. More specifically, an adjusted *p* value according to Benjamini–Hochberg of less than 0.01 and a fold change between 5 and 50 were chosen as the enrichment threshold. Conversion to BED format and manipulation of BED files were carried out using BEDTools [56]. Peak annotation or the identification of genes associated with peaks was performed with PeakAnalyzer [57] according to the Nearest Downstream Gene (NDG) criterion. Specifically, a peak was associated with a gene when it overlapped any of the gene regions or when it was located at most 2 kb upstream of its transcription start site (TSS). A gene was assumed to be marked when at least one peak was found to be associated with it. H2AK121ub- and H3K27me3-marked genes were identified in the WT samples. Each replicate was analyzed separately and the final set of marked genes was determined as those detected in both replicates (Additional file 1: Figure S1).

### Peak visualization

The Integrative Genome Viewer (IGV) [58] was used for peak profile visualization. Read counts were RPKM (reads per kilobase and million mapped reads) normalized using the deepTools [59] utility bamCoverage with a bin size of 10 bp. Scatter plots comparing RPKM normalized peak values for each replicate show high similarity and reproducibility between replicates (Additional file 1: Figures S1, S8, and S12). Metagene plots representing the coverage of H2AK121ub and H3K27me3 marks were generated using the Bioconductor R package ChIPpeakAnno [60] (http://bioconductor.org/packages/release/bioc/html/ChIPpeakAnno.html). The significance of the overlaps between H2AK121ub or H3K27me3 peaks and *A. thaliana* gene regions (obtained from the Bioconductor R package TxDb.Athaliana.BioMart.plantsmart28) was determined using the functions shuffle and enrichPeakOverlap from the R Bioconductor package chipseeker [61]. We generated 500 random shuffles of H2AK121ub or H3K27me3 peaks to estimate the background null distribution of the overlap with the following genomic regions: intergenic, promoter (2 kb upstream of the TSS), 5′ UTR, first exon, gene body, and 3′ UTR. *P* values were corrected for multiple testing using the Benjamini–Hochberg precedure. Percentages of genes showing H2AK121ub and H3K27me3 peaks at annotated genic and intergenic regions in the *A. thaliana* genome were computed using the Bioconductor R package GenomicRanges (http://bioconductor.org/packages/release/bioc/html/GenomicRanges.html). Heatmaps representing the intensity of H2AK121ub and H3K27me3 marks around peak centers were generated using the Bioconductor R package ChIPpeakAnno [61]. RPKM and total library size (reads per million reads sequenced (RPM)) normalizations produced similar qualitative results with a sharper apparent decrease in the case of total library size normalization when comparing *atbmi1a/b/c* to WT (Additional file 1: Figures S13 and S18).

### Transcriptomic analysis by RNA sequencing

In order to analyze the expression levels of marked genes, RNA-seq was performed in two biological replicates for WT and *atbmi1a/b/c* mutant plants at 7 DAG. The Qiagen RNAeasy minikit was used for RNA extraction following the manufacturer’s instructions. RNA concentration and purity were tested using *nanodrop*-photometric quantification (Thermo Scientific). The TruSeq RNA Sample Prep Kit v2 Illumina was used for library preparation following the manufacturer’s recommendations. Sequencing of RNA libraries was carried out with the Illumina HiSeq 2000 sequencer, yielding an average of approximately 15 million 100-nucleotide long paired-end reads for each sample. The high quality of each sample was verified using the software package FASTQC. The number of reads and concurrent pair alignment rate per sequencing sample and scatterplots of pairwise comparison between RNA-seq replicates are shown in Additional file 1: Figure S6. Read mapping to the *A. thaliana* TAIR10 reference genome, transcript assembly, and differential expression were performed with the software tools TopHat andCufflinks [62]. Differentially expressed genes (DEGs) were selected according to the false discovery rate (FDR) calculation performed by cuffdiff, a tool from the cufflinks package. *P* values were corrected for multiple testing using the Benjamini–Hochberg procedure. The Bioconductor R package cummeRbund (http://www.bioconductor.org/) was used for result processing and visualization. An FDR of 0.05 was used for DEG selection. Gene expression was measured in FPKM (fragments per kilobase of exon and million mapped reads). A gene was assumed to be expressed when its FPKM was higher than 5. Differentially expressed genes were selected according to false discovery rate calculation and a log-fold change cut-off >|1| in *atbmi1a/b/c* when compared to Col-0 and a *p* value <0.05.

### Gene Ontology term and transcription factor family enrichment analysis

The R Bioconductor package clusterProfiler [63] was used for Gene Ontology (GO) term enrichment analysis applying the Singular Enrichment Analysis (SEA) algorithm. The list of transcription factor families in *A. thaliana* was downloaded from the plant transcription factor database PlantTFDB 3.0 [64]. Transcription factor family enrichment analysis in the sets of marked genes was performed using Fisher’s exact test (Additional file 1: Figure S7).

### Quantitative comparison of ChIP-seq samples

In order to quantitatively compare ChIP-enriched regions (peaks) detected in WT to those in *atbmi1a/b/c*, *clf28/swn7*, and *lhp1* mutants a variant of the MAnorm [41] approach was taken. MAnorm main assumption states that the true intensities (estimated as read counts) of most commons peaks between the two samples being compared are identical and therefore the detected differences can be used to rescale (using robust linear regression) the intensities of all peaks; however, this does not hold for *atbmi1a/b/c* since the intensities of most common peaks in these mutants are truly affected (Additional file 1: Figure S15) as global levels of H2AK121ub are substantially decreased [24] (Fig. 4). Using all common peaks for rescaling produced a bias that resulted in too few detected peaks with decreased and too many with increased levels in the mutants; for instance, genes like *WUS*, *MAGPIE (MGP) KNUCKLES* (*KNU*), or *WOX12*, which were found to be upregulated in *atbmi1a/b/c* mutants (RNA-seq data), displayed increased levels of H2AK121ub after normalization. We therefore required peaks for which a symmetric distribution was likely to estimate a correction for the entire dataset. The set of common peaks serving as a reference to build the rescaling model for normalization were restricted to those common peaks exhibiting a change of ±20% in RPKM data compared to WT since we found that a reduction of 20% in the levels of H2AK121ub already had a significant impact on gene expression in *atbmi1a/b/c* mutants (Additional file 1: Figure S16). For the comparison between *atbm1a/b/c* and the WT we therefore constrained the set of peaks used for normalization to those whose associated genes were not differentially expressed. Moreover, a high rate of non-significant changes were present in the peaks with a variation smaller than 20% whereas peaks with a reduction greater than 20% exhibited a high rate of significant changes (Additional file 1: Figure S16). Applying this modification, we found that *WUS*, *MGP*, *KNU*, and *WOX12* displayed decreased levels (Additional file 5: Dataset S4), which is in line with ChIP-qPCR results (Additional file 1: Figure S16), expression analysis, and previously published results [24]. Additional file 1: Figures S10, S15 and S19 show the selected common peaks used for building the rescaling model and the fold change (M)/mean intensities (A) of all peaks after normalization.

For the selection of differential peaks in *atbmi1a/b/c*, *clf28/swn7*, and *lhp1* compared to WT, each replicate was analyzed separately. An adjusted *p* value cutoff of 0.05 was used and peaks were classified into different groups. Peaks exhibiting less than 80% of the WT intensity were assumed to have differentially reduced their intensity whereas peaks exhibiting more than 120% of the WT intensity were assumed to have differentially increased their intensity. The final set of differential peaks was taken as the intersection between the differential peaks found in each replicate.

The annotation of differential peaks was performed with PeakAnalyzer using the NDG criterion as described previously. When several peaks were found to be associated with a gene, only the one exhibiting the biggest decrease in the observed mark was taken into account.

### Significance of Venn diagram overlaps

The significance of Venn diagram overlaps was analyzed using Fisher’s exact test. Specifically, the function fisher.test from the R package stats was used.

### Western blot analysis

An aliquot of fixed chromatin after sonication was boiled for 10 min in SDS-PAGE buffer. Proteins were separated on 12% SDS-PAGE gel and transferred to a PVDF membrane (Immobilon-P Transfer membrane, Millipore) by semi-dry blotting in 25 mM Tris–HCl, 192 mM glycine, and 10% methanol. The following antibodies were used: anti-H3K27me3 polyclonal antibody (Diagenode, C15410069), anti-H2AUb (Cell-Signalling Technology, 8240S), and anti-H3 (Agrisera, AS10 710). Horseradish peroxidase-conjugated goat anti-rabbit antibody (Sigma-Aldrich, A0545) was used as secondary antibody at 1/10,000 dilution. Chemiluminescence detection was performed with ECL Prime Western Blotting Detection Reagent (GE Healthcare Life Sciences) following the manufacturer’s instructions.

## Additional files

Additional file 1: Supplementary Tables and Figures. **Table S1**. Total and mapped reads produced for each ChIP-seq sample. **Table S2**. Primers used for ChIP qPCR. **Figure S1**. Comparison of H2AK121ub and H3K27me3 ChIP-seq replicates in WT Arabidopsis seedlings at 7 DAG. **Figure S2**. Identification of H2AK121ub peaks. **Figure S3**. Genome wide localization of H2AK121ub and H3K27me3 marks in Arabidopsis WT seedlings at 7 DAG. **Figure S4**. ChIP-qPCR validations of H2AK121ub levels in WT at 7 DAG. **Figure S5**. Genomic distribution of H2AK121ub marks at different categories of marked genes. **Figure S6**. RNA-seq analysis of WT and atbmi1a/b/c at 7 DAG. **Figure S7**. Different families of Transcription Factors (TFs) are enriched in H2AK121ub/H3K27me3 and only-H3K27me3 marked genes. **Figure S8**. Comparison of clf28/swn7 and lhp1 H2AK121ub ChIP-seq replicates at 7 DAG . **Figure S9**. The global levels of H2AK121ub marks are increased in clf28/swn7 mutants. **Figure S10**. H2AK121ub levels at H2AK121ub peaks in clf28/swn7 and lhp1 mutants compared to WT after normalization with a modified MAnorm protocol. **Figure S11**. ChIP-qPCR validations of H2AK121ub levels in clf28/swn7 at 7 DAG. **Figure S12**. Comparison of atbmi1a/b/c ChIP-seq replicates at 7 DAG . **Figure S13**. The global levels of H2AK121ub marks are reduced in atbmi1a/b/c mutants. **Figure S14**. H2AK121ub levels at selected genes in WT and atbmi1a/b/c. **Figure S15**. H2AK121ub levels at H2AK121ub peaks in atbmi1a/b/c mutant compared to WT after normalization with a modified MAnorm protocol. **Figure S16**. Genes displaying reduced levels of H2AK121ub marks were significantly enriched in genes that are transcriptionally upregulated in atbmi1a/b/c mutants. **Figure S17**. Loss of AtBMI1 function impacts H2AK121ub levels. **Figure S18**. ChIP-seq density heatmaps of H3K27me3 marks in WT and atbmi1a/b/c mutants at genomic regions surrounding the TSS of target genes. **Figure S19**. H3K27me3 levels at H3K27me3 peaks in atbmi1a/b/c mutant compared to WT after normalization with a modified MAnorm protocol. **Figure S20**. Loss of AtBMI1 function impacts H3K27me3 levels. (PDF 1398 kb)

Additional file 2: Supplementary Dataset S1: Expression levels of Genes marked only-H2AK121ub, H2AK121ub/H3K27me3 and only-H3K27me3 marks. (XLSX 747 kb)

Additional file 3: Supplementary Dataset S2: Genes exhibiting changes of H2AK121ub in clf28/swn7. (XLSX 99 kb)

Additional file 4: Supplementary Dataset S3: Genes exhibiting changes in H2AK121ub in lhp1. (XLSX 50 kb)

Additional file 5: Supplementary Dataset S4: Genes exhibiting changes in H2AK121ub in atbmi1a/b/c. (XLSX 415 kb)

Additional file 6: Supplementary Dataset S5: Genes exhibiting changes in H3K27me3 in atbmi1a/b/c. (XLSX 180 kb)
