# Supplementary material for: H2A monoubiquitination in Arabidopsis thaliana is generally independent of LHP1 and PRC2 activity
Source: Genome Biol. 2017 Apr 12;18:69. doi: 10.1186/s13059-017-1197-z (PMC5389094; doi:10.1186/s13059-017-1197-z)
Supplement: Supplementary file 1 — Supplementary Tables and Figures. Table S1. Total and mapped reads produced for each ChIP-seq sample. Table S2. Primers used for ChIP qPCR. Figure S1. Comparison of H2AK121ub and H3K27me3 ChIP-seq replicates in WT Arabidopsis seedlings at 7 DAG. Figure S2. Identification of H2AK121ub peaks. Figure S3. Genome wide localization of H2AK121ub and H3K27me3 marks in Arabidopsis WT seedlings at 7 DAG. Figure S4. ChIP-qPCR validations of H2AK121ub levels in WT at 7 DAG. Figure S5. Genomic distribution of H2AK121ub marks at different categories of marked genes. Figure S6. RNA-seq analysis of WT and atbmi1a/b/c at 7 DAG. Figure S7. Different families of Transcription Factors (TFs) are enriched in H2AK121ub/H3K27me3 and only-H3K27me3 marked genes. Figure S8. Comparison of clf28/swn7 and lhp1 H2AK121ub ChIP-seq replicates at 7 DAG . Figure S9. The global levels of H2AK121ub marks are increased in clf28/swn7 mutants. Figure S10. H2AK121ub levels at H2AK121ub peaks in clf28/swn7 and lhp1 mutants compared to WT after normalization with a modified MAnorm protocol. Figure S11. ChIP-qPCR validations of H2AK121ub levels in clf28/swn7 at 7 DAG. Figure S12. Comparison of atbmi1a/b/c ChIP-seq replicates at 7 DAG . Figure S13. The global levels of H2AK121ub marks are reduced in atbmi1a/b/c mutants. Figure S14. H2AK121ub levels at selected genes in WT and atbmi1a/b/c. Figure S15. H2AK121ub levels at H2AK121ub peaks in atbmi1a/b/c mutant compared to WT after normalization with a modified MAnorm protocol. Figure S16. Genes displaying reduced levels of H2AK121ub marks were significantly enriched in genes that are transcriptionally upregulated in atbmi1a/b/c mutants. Figure S17. Loss of AtBMI1 function impacts H2AK121ub levels. Figure S18. ChIP-seq density heatmaps of H3K27me3 marks in WT and atbmi1a/b/c mutants at genomic regions surrounding the TSS of target genes. Figure S19. H3K27me3 levels at H3K27me3 peaks in atbmi1a/b/c mutant compared to WT after normalization with a modified MAnorm pr [file 13059_2017_1197_MOESM1_ESM.pdf]

Table S1. Total and mapped reads produced for each ChIP-seq sample

Sample	Total Reads	Mapped Reads
Col-0 H3K27me3 rep1	12050662	10334323 (85.76%)
Col-0 H3K27me3 rep2	9176437	7986731 (87.04%)
atbmi1a/b/c H3K27m3 rep1	10887806	7986731 (87.04%)
atbmi1a/b/c H3K27m3 rep2	10013720	8320393 (83.09%)
clf28/swn7 H3K27me3 rep1	9831362	5195906 (52.85%)
clf28/swn7 H3K27me3 rep2	15239663	8506182 (55.82%)
Col-0 H2AK121ub rep1	17823921	15895954 (89.18%)
Col-0 H2AK121ub rep2	17210488	15531618 (90.25%)
atbmi1a/b/c H2AK121ub rep1	14700283	8355733 (56.84%)
atbmi1a/b/c H2AK121ub rep2	17587127	11734768 (66.72%)
Col-0 H2AK121ub rep1	12319965	10756227 (87.31%)
Col-0 H2AK121ub rep2	12068263	9791317 (81.13%)
clf28/swn7 H2AK121ub rep1	13698520	11708105 (85.47%)
clf28/swn7 H2AK121ub rep2	13315004	12010139 (90.20%)
Col-0 H2AK121ub rep1	21853396	15824831 (72.41%)
Col-0 H2AK121ub rep2	19184893	16335871 (85.15%)
lhp1 H2AK121ub rep1	25534779	20282810 (79.43%)
lhp1 H2AK121ub rep2	20734198	16660391 (80.35%)
Input	43489511	31200705 (71.74%)

Table S2. Primers used for ChIP qPCR

ChIP primers (5´- 3´)	
ABCG35 Fw	*GATGGATTACGATCCAGCTCAT*
ABCG35 Rv	*CTGCTTGCTTTGCTTACACTAC*
TOUCH4 Fw	*ACCACAAACCAATCTAACTCAATAC*
TOUCH4 Rv	*CAGAGGAGGTGATGATAAGAGAAA*
FBA2 Fw	*TTCCTCCTACGCCGATGA*
FBA2 Rv	*AAACACGTGGTAGAGAAGAGA*
FAD3 Fw	*GGAGACCGGAAGAAAGAAGAAA*
FAD3 Rv	*CTTAACCCAACAGTGCTTAGGA*
AIL5 Fw	*GTTGTCTTCTCTTCCGACCTAC*
AIL5 Rv	*CTAGGAAGCTAGCGGCTATTG*
FLC Fw	*CGTACTTATCGCCGGAGGAG*
FLC Rv	*CATCGAGAAAGCTCGTCAGC*
GA3OX1 Fw	*GGTGCCTTCCAAATCTCAAAC*
GA3OX1 Rv	*GAAGAGACTACCGGTGAGAAAC*
KNU Fw	*AGTTCTACACATCTCAAGCTCTC*
KNU Rv	*GCAAGGGTAAGGACGAAGAA*
SPL8 Fw	*CCAACAGCAACAACCAGTTTC*
SPL8 Rv	*GGATGGTAGATGGGATCGGA*
DPA4 Fw	*GACACGTCATCTTCGGAGAAA*
DPA4 Rv	*TTTGACTGCTGTTCCAGTAAGA*
SUP Fw	*CTTTCACAGTCTTAACACCAAAGAG*
SUP Rv	*GAGAGAGAGAGAGAGATATAGAGATGAG*
CAL Fw	*GCACTCTTTCACATTACCATCATTAG*
CAL Rv	*CCTCTTCAATTCAACCCTACCC*
SEP3 Fw	*CCTATGAGGGTCTTTGGTACAC*
SEP3 Rv	*ACATGTCAGCTTCATTACTTGA*
MGP Fw	*CTACCGACCAATCAGCGTATC*
MGP Rv	*CGATCTCGTGAAGACCGTTATTA*
WUS Fw	*CCACAGCATCAGCATCATCA*
WUS Rv	*GACACGTGTAACCACCAGAG*
WOX12 Fw	*TGGATCGTCGTCATCTCAAATC*
WOX12 Rv	*GATTCTCCATACCATTGTTGTTCTC*

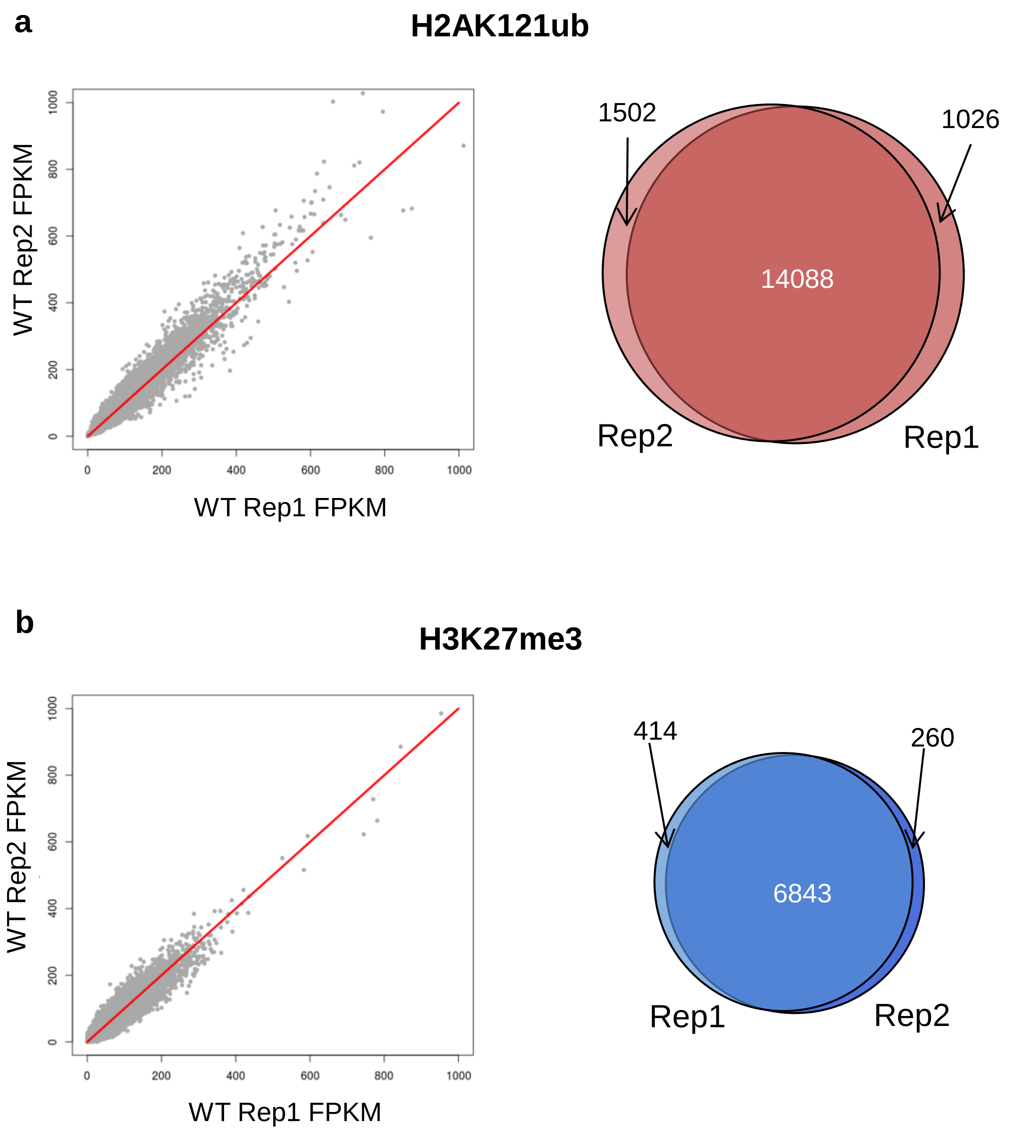

Figure S1. Comparison of H2AK121ub and H3K27me3 ChIP-seq replicates in WT Arabidopsis seedlings at 7 DAG. (a) Scatterplot of pairwise comparison between H2AK121ub ChIP-seq replicates (left panel) and Venn diagram showing overlapping H2AK121ub marked genes between replicates (right panel). **(b)** Scatterplot of pairwise comparison between H3K27me3 ChIP-seq replicates (left panel) and Venn diagram showing overlapping H3K27me3 marked genes between replicates (right panel).

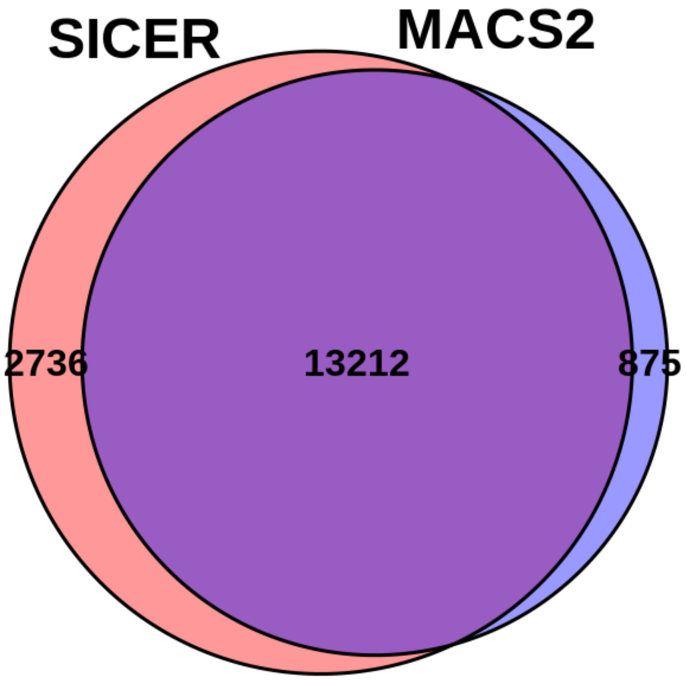

Figure S2. Identification of H2AK121ub peaks. Two different detection methods, MACS and SICER, were coincident in 93.79 % of the identified H2AK121ub peaks

a

b

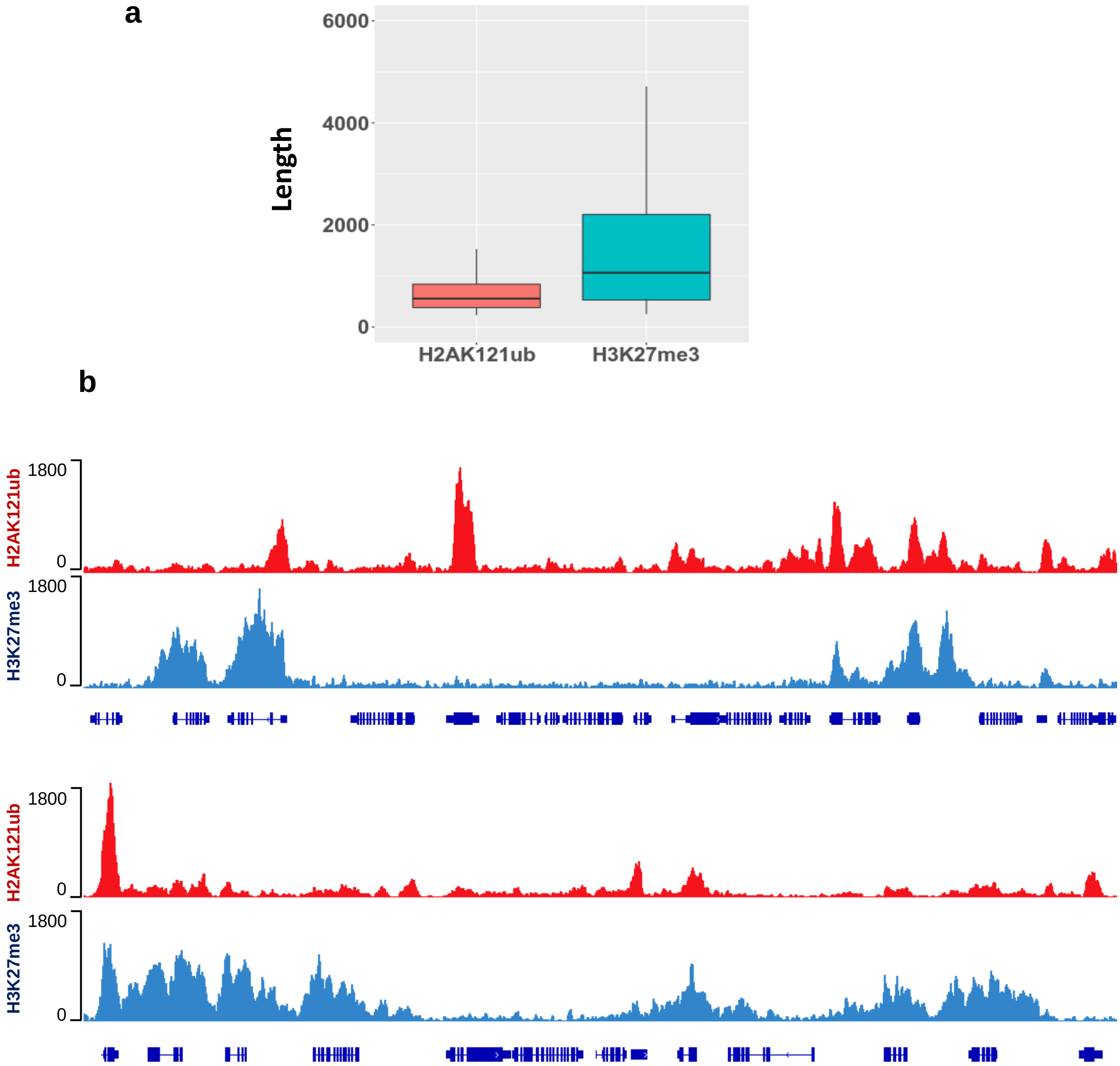

Figure S3. Genome wide localization of H2AK121ub and H3K27me3 marks in Arabidopsis WT seedlings at 7 DAG. (a) Boxplots representing length distribution of H2AK121ub and H3K27me3 peaks. H3K27me3 peaks have an average length of 1.7 Kb, while H2AK121ub peaks were sharper with an average length of 0.6 Kb. This difference is significant according to a p-value of 2.2x10-16 obtained using the Wilcoxon test. **(b)** Representative genome browser views of H2AK121ub and H3K27me3 peaks.

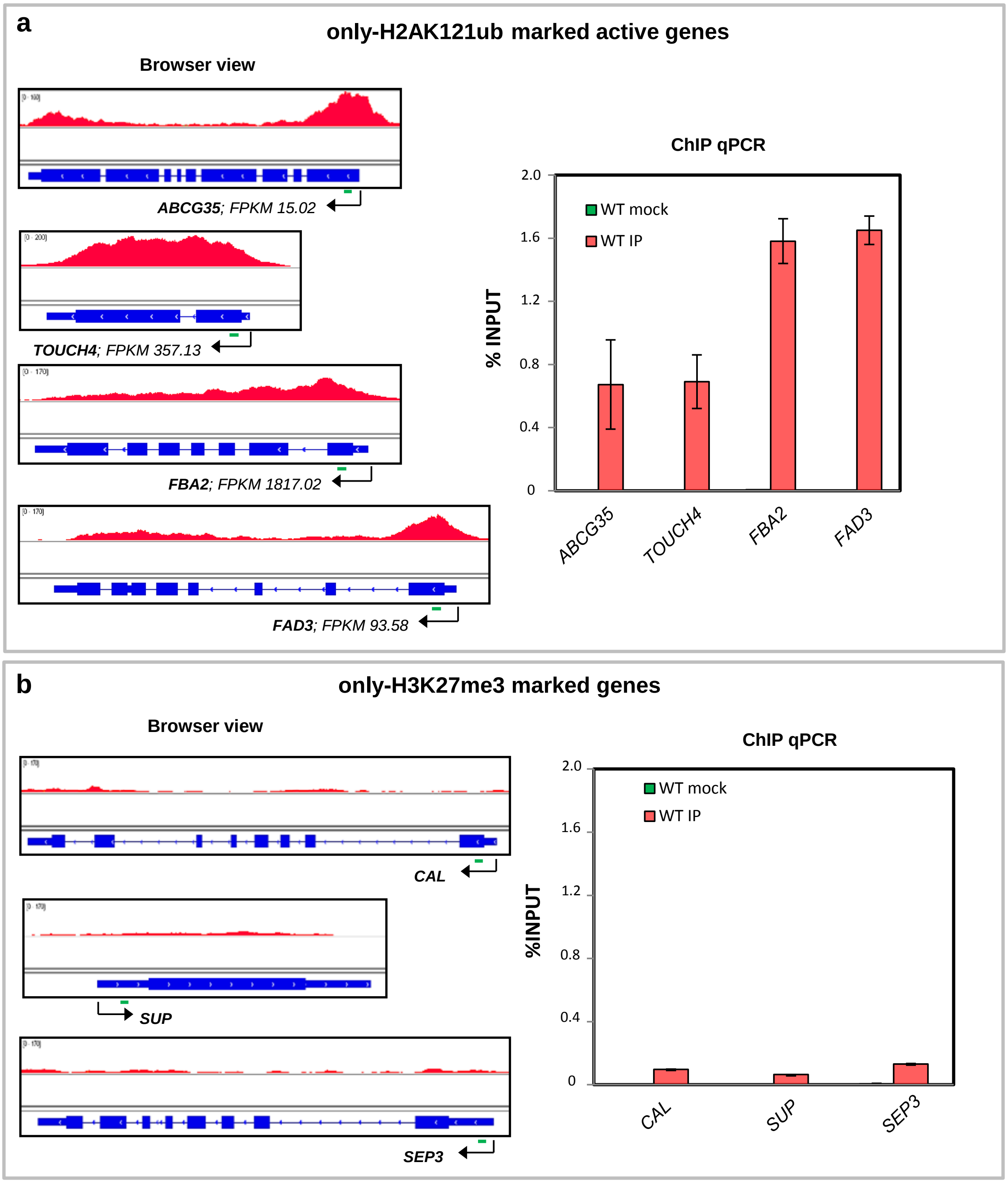

Figure S4. ChIP-qPCR validations of H2AK121ub levels in WT at 7 DAG. (a) H2AK121ub levels at only-H2AK121ub marked active genes. The expression levels of the genes are indicated in FPKM. **(b)** H2AK121ub levels at only-H3K27me3 marked genes. Structure of the genes and location of the region amplified by qPCR (green line) are shown in the Browser views. For each locus, the amount of immunoprecipitated DNA using H2AK121ub antibody (IP) or no antibody (mock) is indicated as % of input. Error bars represent SD between replicates.

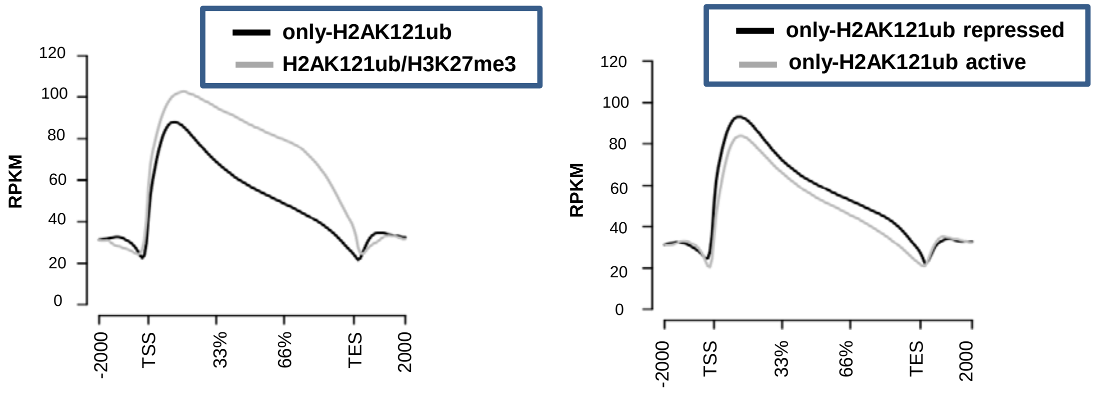

Figure S5. Genomic distribution of H2AK121ub marks at different categories of marked genes. **(a)** Metagene plot showing the distribution of H2AK121ub marks at only-H2AK121ub and H2AK121ub/H3K27me3 marked genes. **(b)** Metagene plot showing the distribution of H2AK121ub marks at only-H2AK121ub active and repressed genes.

a

Sample	Total Number of Reads	Concurrent Pair Alignment Rate
**WT rep1**	57837872	51421443 (88.7%)
**WT rep2**	49749961	43185550 (86.8%)
**atbmi1a/b/c rep1**	54627448	48458316 (88.6%)
**atbmi1a/b/c rep2**	46742567	40695811 (87.0%)

b

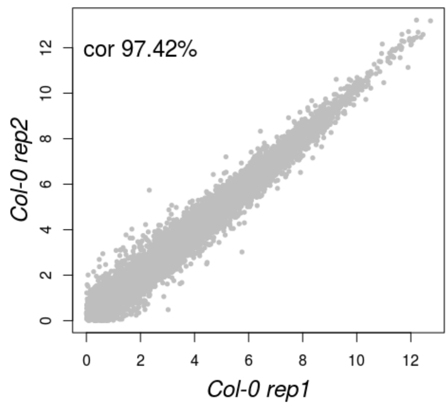

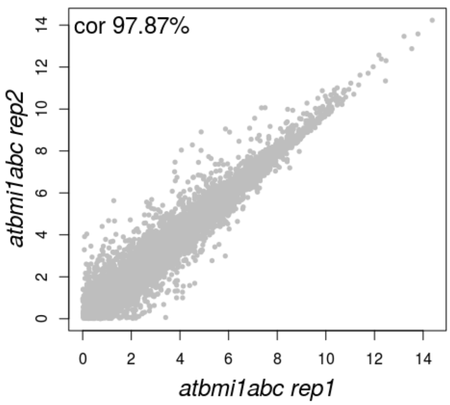

Figure S6. RNA-seq analysis of WT and *atbmi1a/b/c* at 7 DAG. (a) Number of reads and concurrent pair alignment rate per sequencing sample. The numbers indicate a high read sequencing quality and the lack of sample contamination. **(b)** Scatterplots of pairwise comparison between RNA-seq replicates of WT (Col-0, left panel) and *atbmi1a/b/c* (right panel).

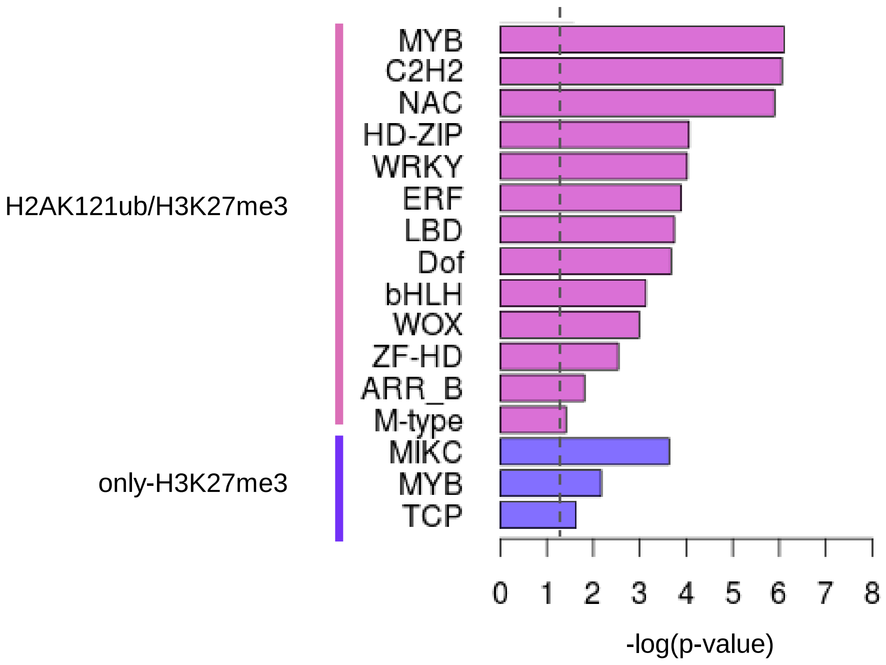

Figure S7. Different families of Transcription Factors (TFs) are enriched in H2AK121ub/H3K27me3 and only-H3K27me3 marked genes. While H2AK121ub/H3K27me3 marked genes showed enrichment for different TF families, only-H3K27me3 marked genes were significantly enriched for MADS-box transcription factors of the MIKC type, which are mostly involved in the control of flowering time, flower, seed, and fruit development. A p-value of 0.05 was used as a cut-off to determine significance (indicated as dashed line). X-axis of the plot represents the significance level using -log(p-value). The p-value was computing using Fisher's Exact test.

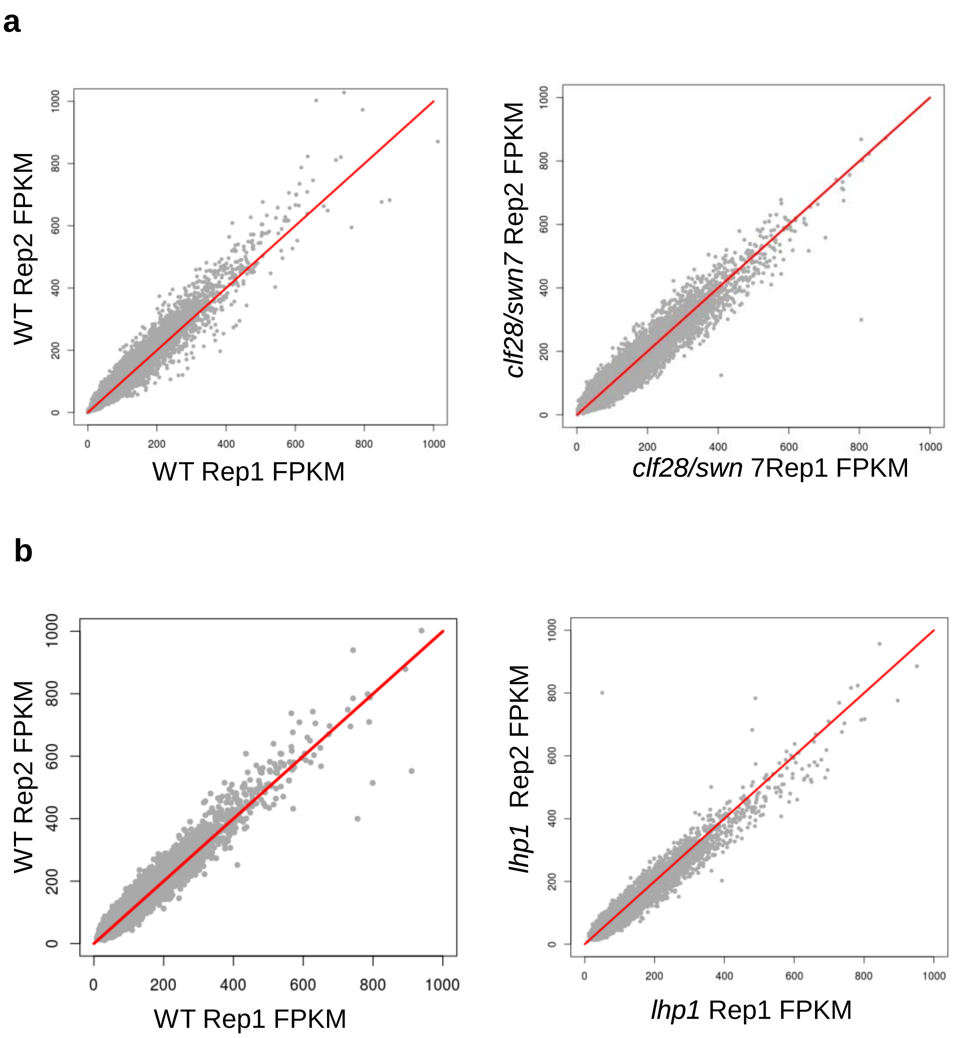

Figure S8. Comparison of *clf28/swn7* and *lhp1* H2AK121ub ChIP-seq replicates at 7 DAG. (a) Scatterplots of pairwise comparison between H2AK121ub ChIP-seq replicates of WT (left panel) and *clf28/swn7* (right panel). **(b)** Scatterplots of pairwise comparison between H2AK121ub ChIP-seq replicates of WT (left panel) and *lhp1* (right panel).

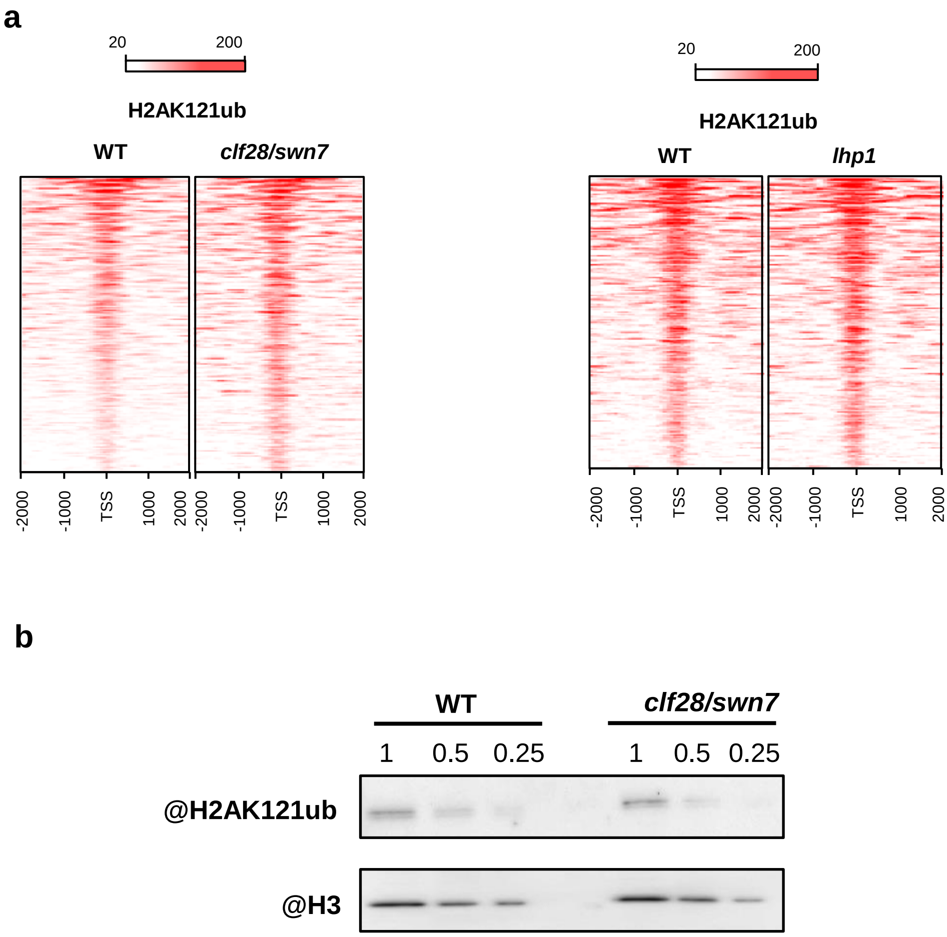

Figure S9. **The global levels of H2AK121ub marks are increased in *clf28/swn7* mutants. (a)** ChIP-seq density heatmaps of H2AK121ub marks in WT and *clf28/swn7* (left panel) and WT and *lhp1* (right panel) mutants at genomic regions surrounding the TSS of target genes. **(b)** Western blot (WB) analysis showing global H2AK121ub levels in WT and *clf28/swn7* mutants at 7 DAG. A two fold dilution series of extracted chromatin before immunoprecipitation was probed with the indicated antibodies to ensure quantitative results.

a

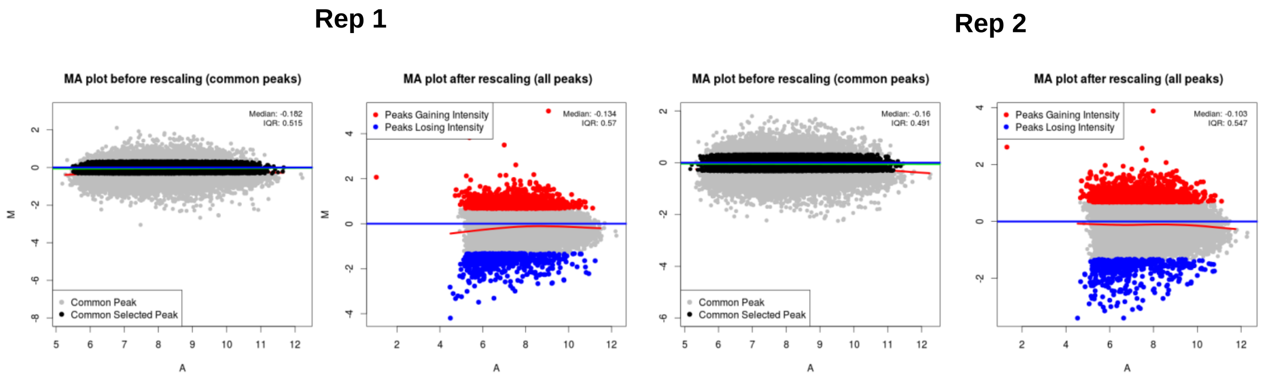

b

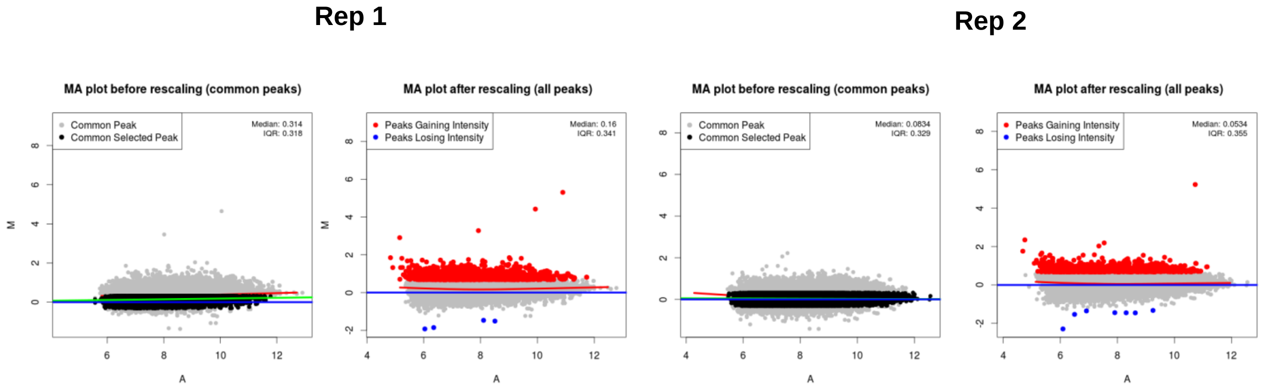

Figure S10. H2AK121ub levels at H2AK121ub peaks in *clf28/swn7* and *lhp1* mutants compared to WT after normalization with a modified MAnorm protocol. **(a,b)** Fold-change (M) and average intensity (A) for common H2AK121ub peaks before normalization and for all peaks after normalization in the comparison between **(a)** *clf28/swn7* and WT and **(b)** *lhp1* and WT. Common peaks used to build the rescaling model are highlighted in black. Peaks with increased levels of H2AK121ub were indicated in red and with decreased levels in blue.

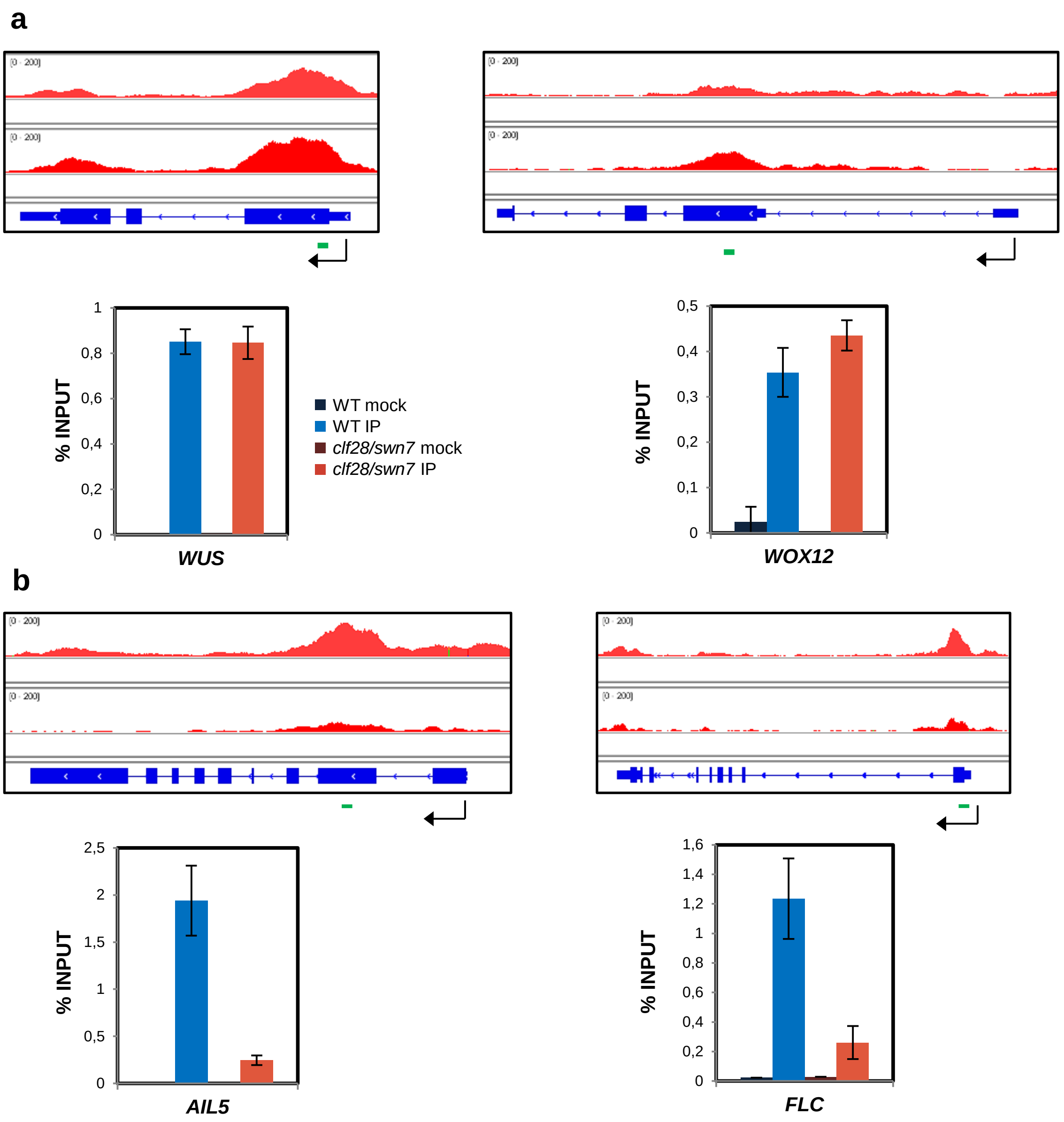

Figure S11. ChIP-qPCR validations of H2AK121ub levels in *clf28/swn7* at 7 DAG. (a) Genes showing unaltered or increased levels of H2AK121ub marks in *clf28/swn7*. **(b)** Genes showing decreased levels of H2AK121ub marks in *clf28/swn7*. Structure of the genes and location of the region amplified by qPCR (green line) are shown in the Browser views. For each locus, the amount of immunoprecipitated DNA using H2AK121ub antibody (IP) or no antibody (mock) is indicated as % of input. Error bars represent SD between replicates.

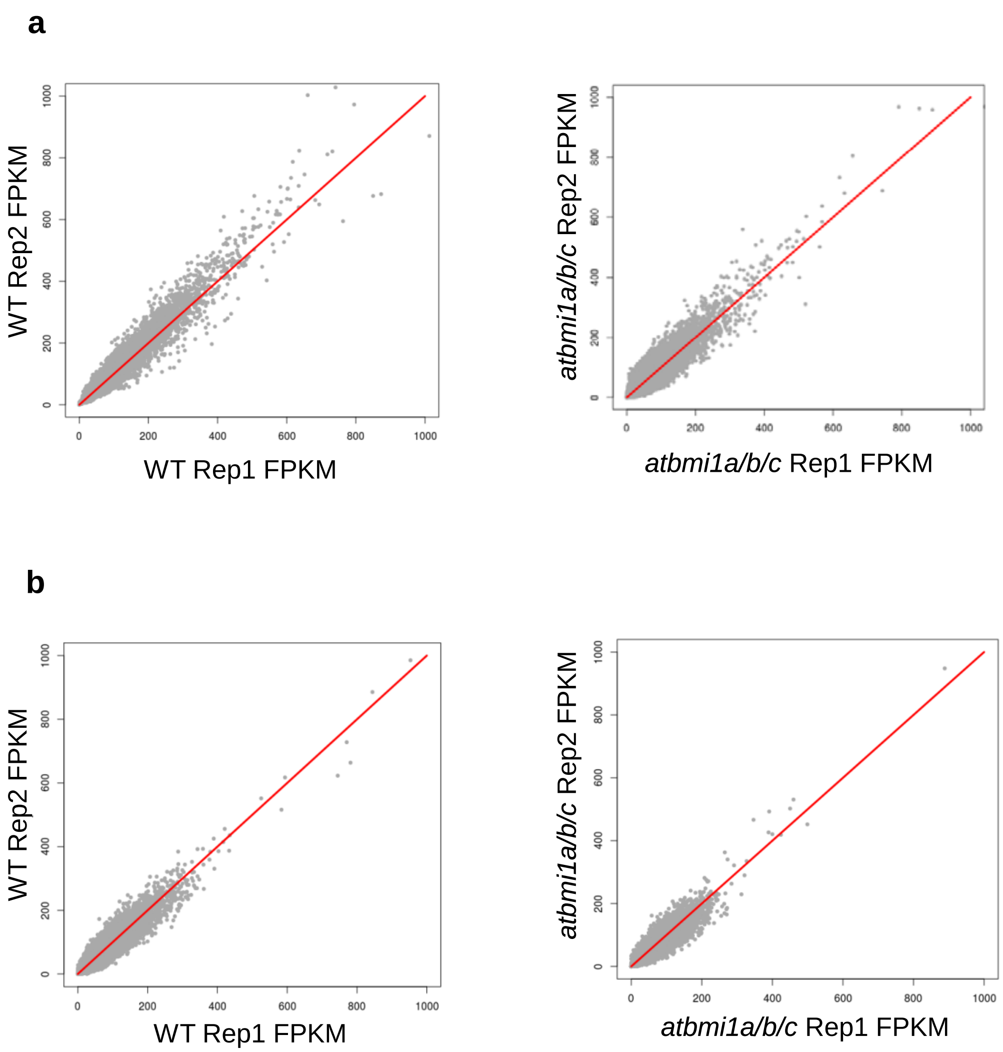

Figure S12. Comparison of *atbmi1a/b/c* ChIP-seq replicates at 7 DAG. (a) Scatterplots of pairwise comparison between H2AK121ub ChIP-seq replicates of WT (left panel) and *atbmi1a/b/c* (right panel). **(b)** Scatterplots of pairwise comparison between H3K27me3 ChIP-seq replicates of WT (left panel) and *atbmi1a/b/c* (right panel).

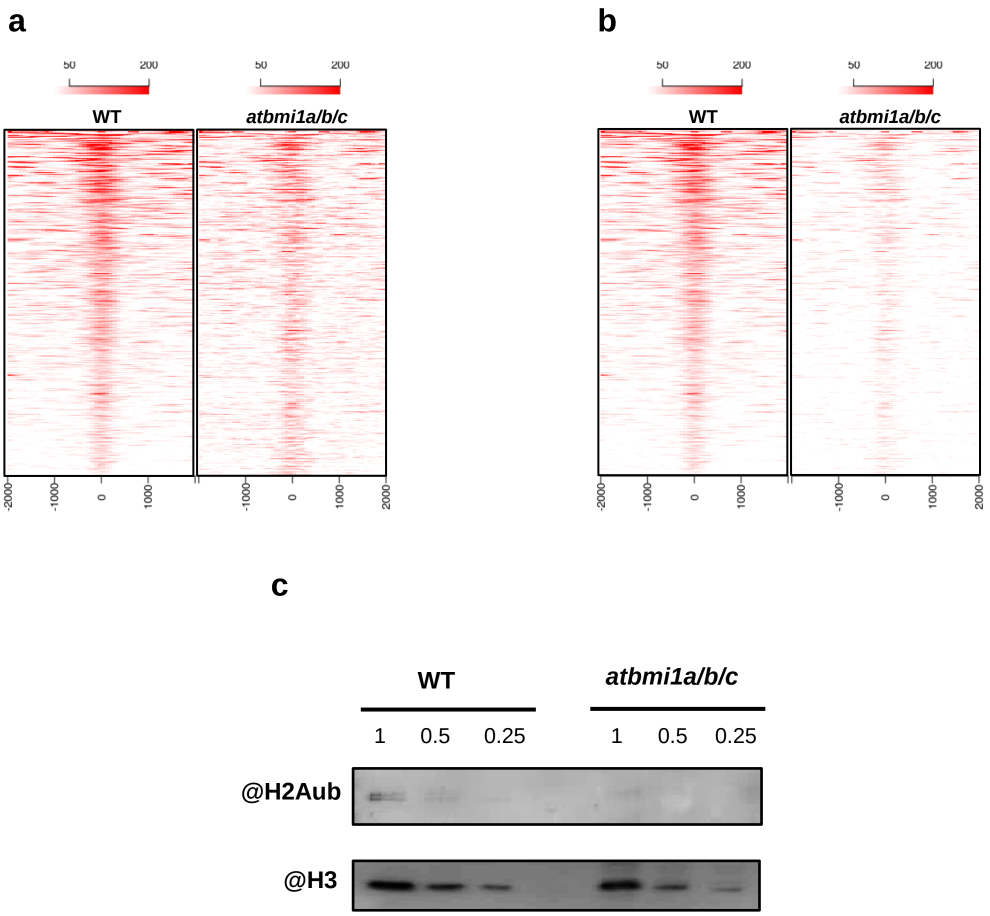

Figure S13. The global levels of H2AK121ub marks are reduced in *atbmi1a/b/c* mutants. (a, b) ChIP-seq density heatmaps of H2AK121ub marks in WT and *atbmi1a/b/c* mutants at genomic regions surrounding the TSS of target genes. **(a)** reads per kilobase and million mapped reads (RPKM) and **(b)** total library size (reads per million reads sequenced (RPM)) normalizations produced similar results with a sharper apparent decrease in the case of total library size normalization when comparing *atbmi1a/b/c* to WT. **(c)** WB analysis showing global levels of H2AK121ub in *atbmi1a/b/c* mutants compared to WT. A two fold dilution series of extracted chromatin before immunoprecipitation was probed with the indicated antibodies.

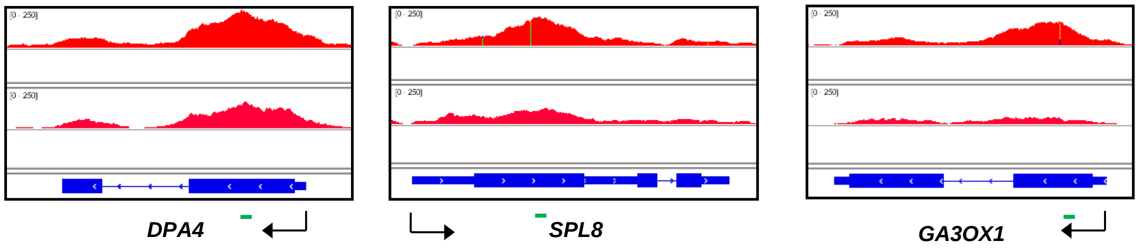

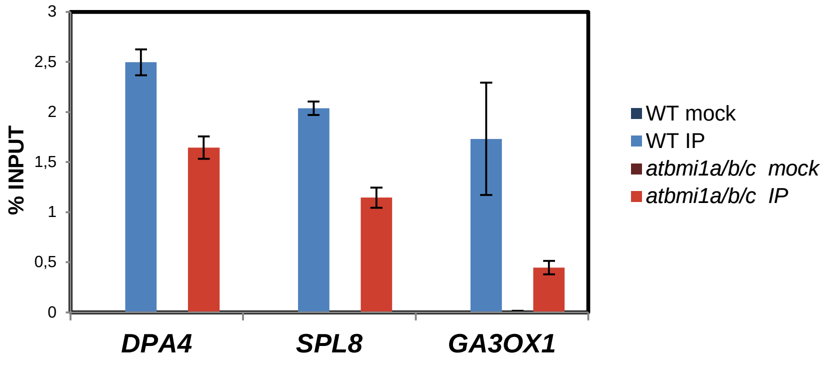

Figure S14. H2AK121ub levels at selected genes in WT and *atbmi1a/b/c*. ChIP qPCR analysis of H2AK121ub levels at genes in WT and *atbmi1a/b/c* mutants. Structure of the genes and location of the region amplified by qPCR (green line) are shown in the Browser views. For each locus, the amount of immunoprecipitated DNA using H2AK121ub antibody (IP) or no antibody (mock) is indicated as % of input. Error bars represent SD between replicates.

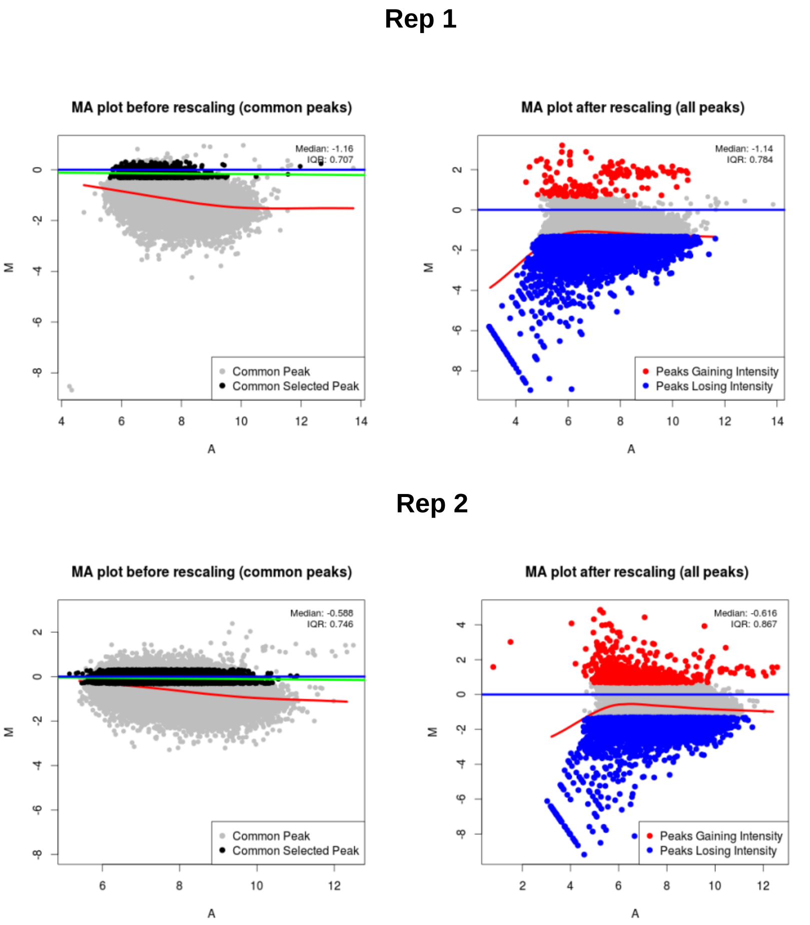

Figure S15. H2AK121ub levels at H2AK121ub peaks in *atbmi1a/b/c* mutant compared to WT after normalization with a modified MAnorm protocol. Fold-change (M) and average intensity (A) for common H2AK121ub peaks before normalization and for all peaks after normalization in the comparison between *atbmi1a/b/c* and WT. Common peaks used to build the rescaling model are highlighted in black. Peaks with increased levels of H2AK121ub were indicated in red and with decreased levels in blue.

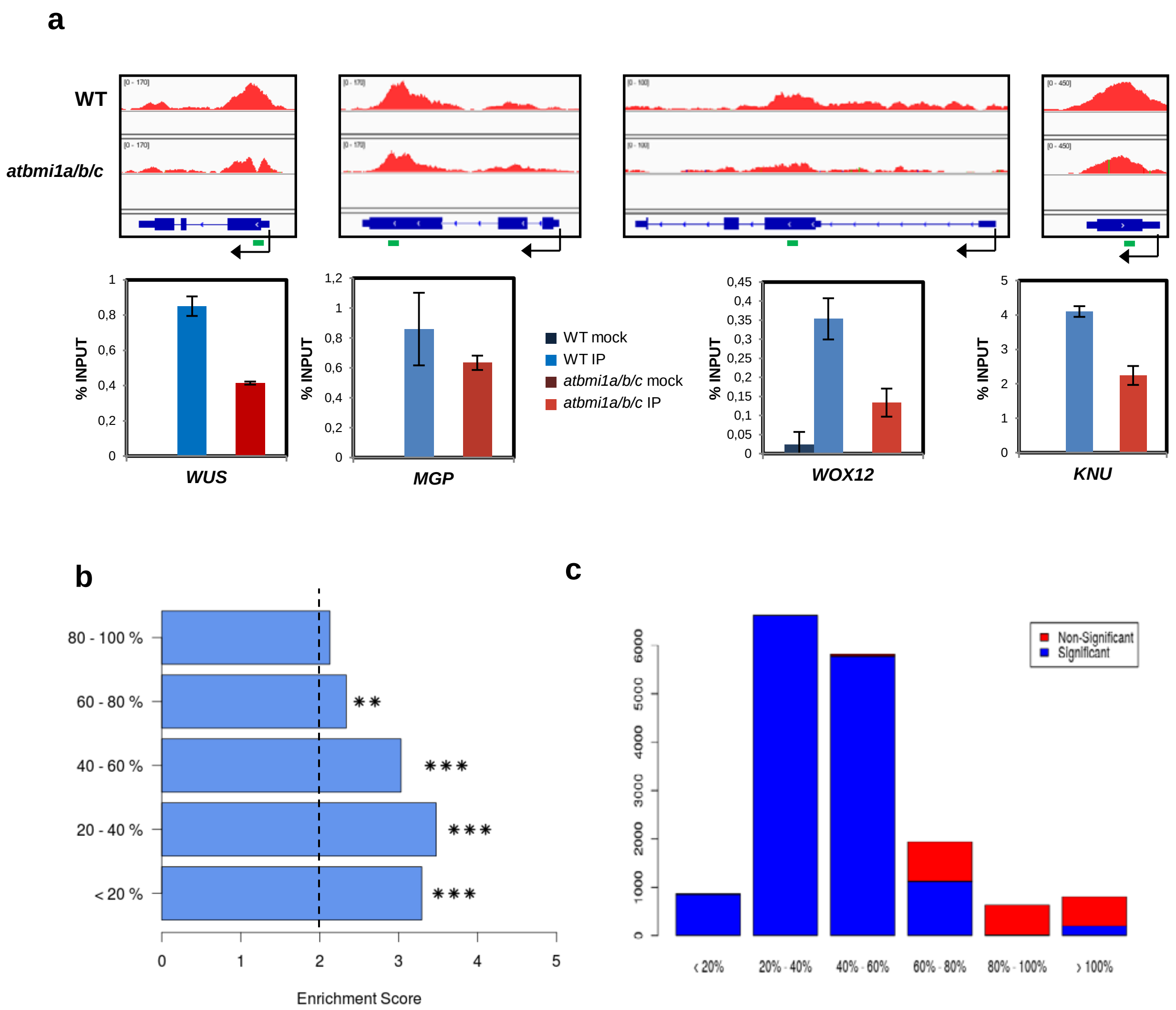

Figure S16. Genes displaying reduced levels of H2AK121ub marks were significantly enriched in genes that are transcriptionally upregulated in *atbmi1a/b/c* mutants. (a) ChIP qPCR analysis of H2AK121ub levels at selected genes in WT and *atbmi1a/b/c* mutants. Structure of the genes and location of the region amplified by qPCR (green line) are shown in the Browser views. For each locus, the amount of immunoprecipitated DNA using H2AK121ub antibody (IP) or no antibody (mock) is indicated as % of input. Error bars represent SD between replicates. **(b)** Enrichment score of upregulated genes in the different gene categories according to their level of change in H2AK121ub between WT and *atbmi1a/b/c* mutants. Genes displaying a reduction of H2AK121ub levels equal or bigger than 20% were significantly enriched in genes that became activated in *atbmi1a/b/c* mutants, indicating that a reduction of 20% may already have an impact on gene expression. **(c)** Fraction of genes in the different categories according to their levels of H2AK121ub marks in *atbmi1a/b/c* mutants that were statistically significant ($p<0.05$ computed by MAnorm based on a Bayesian model [42]).

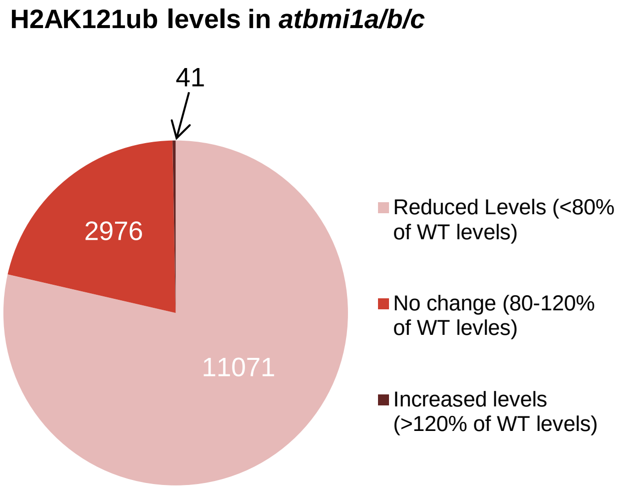

Figure S17. Loss of AtBMI1 function impacts H2AK121ub levels. Pie chart showing the total number of genes with altered levels of H2AK121ub in *atbmi1a/b/c* mutants at 7 DAG. 78% of H2AK121ub marked genes displayed reduced levels of these marks in *atbmi1a/b/c* mutants, ranging from 0 to 80% of WT levels.

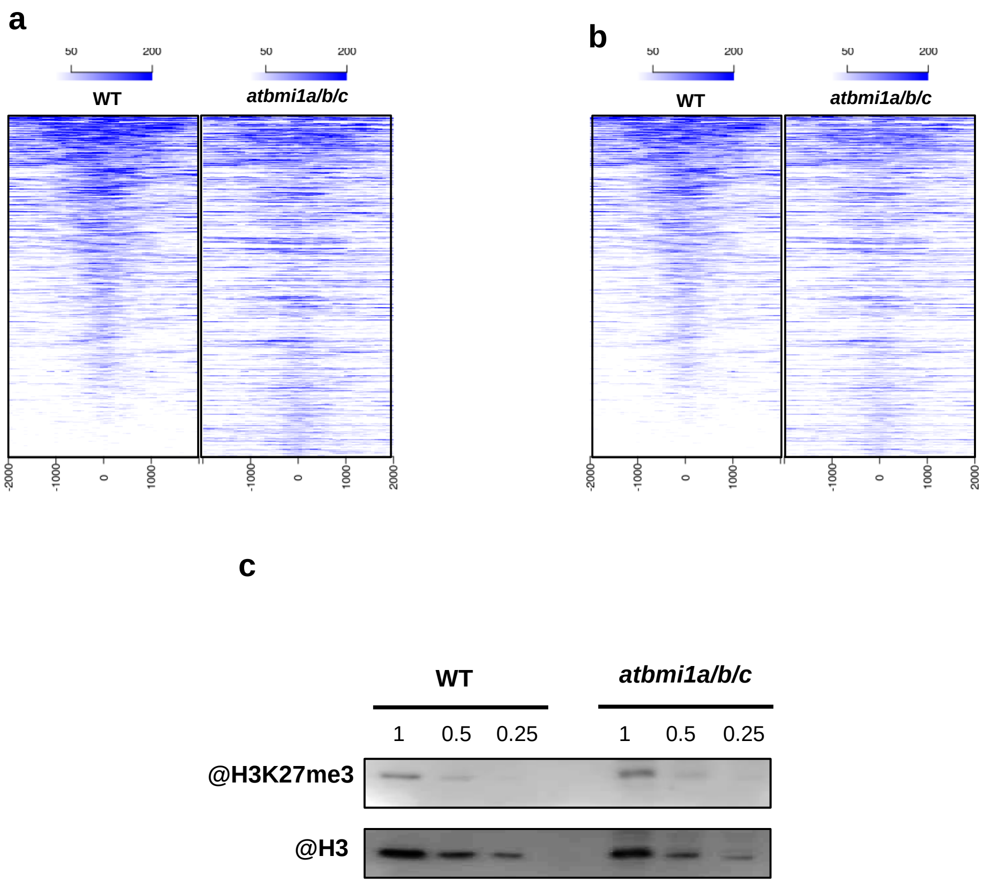

Figure S18. ChIP-seq density heatmaps of H3K27me3 marks in WT and *atbmi1a/b/c* mutants at genomic regions surrounding the TSS of target genes. **(a)** reads per kilobase and million mapped reads (RPKM) and **(b)** total library size (reads per million reads sequenced (RPM)) normalizations produced similar results with a sharper apparent decrease in the case of total library size normalization when comparing *atbmi1a/b/c* to WT. **(c)** WB analysis showing global levels of H3K27me3 in *atbmi1a/b/c* mutants compared to WT. A two fold dilution series of extracted chromatin before immunoprecipitation was probed with the indicated antibodies.

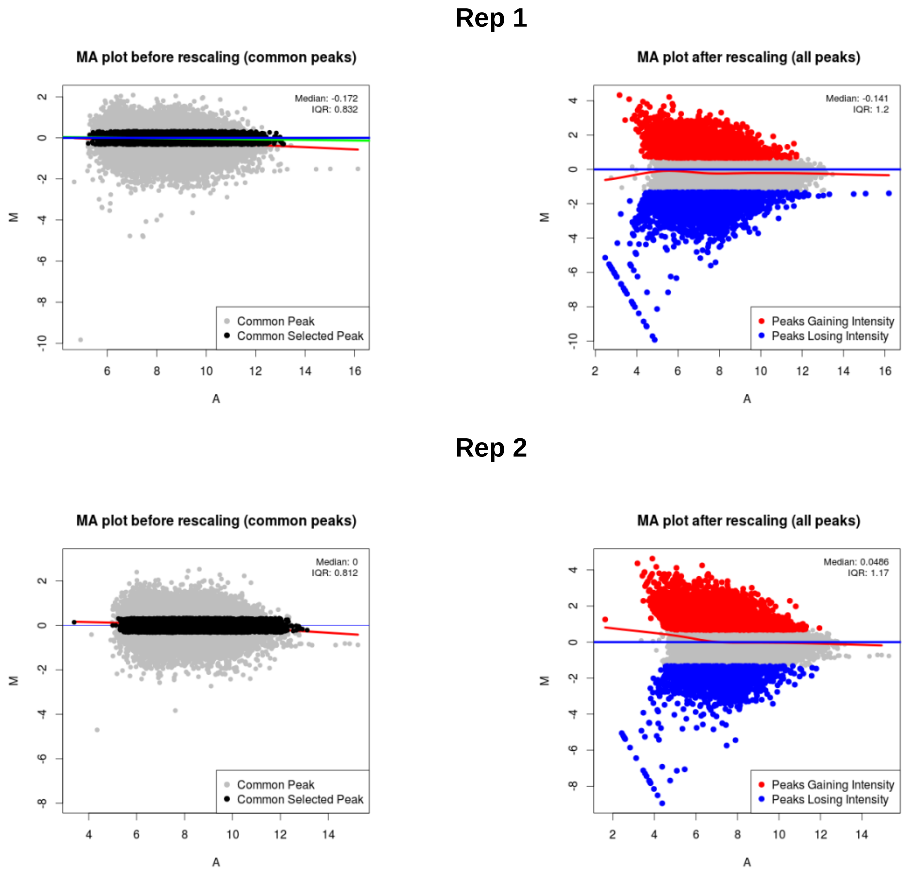

Figure S19. H3K27me3 levels at H3K27me3 peaks in *atbmi1a/b/c* mutant compared to WT after normalization with a modified MAnorm protocol. Fold-change (M) and average intensity (A) for common H3K27me3 peaks before normalization and for all peaks after normalization in the comparison between *atbmi1a/b/c* and WT. Common peaks used to build the rescaling model are highlighted in black. Peaks with increased levels of H3K27me3 were indicated in red and with decreased levels in blue.

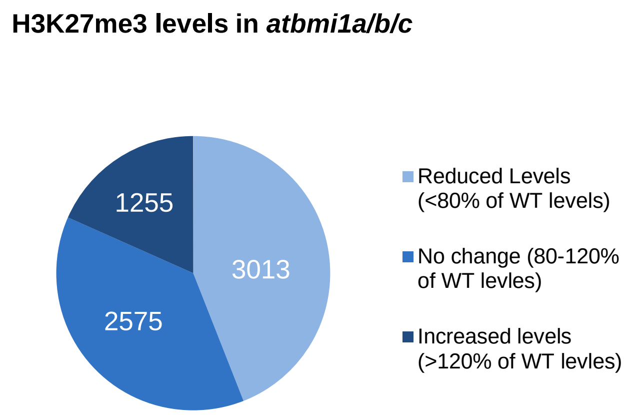

Figure 20. Loss of AtBMI1 function impacts H3K27me3 levels. Pie chart showing the total number of genes with altered levels of H3K27me3 in *atbmi1a/b/c* mutants at 7 DAG. 44% of H2AK121ub marked genes displayed reduced levels of these marks in *atbmi1a/b/c* mutants, ranging from 0 to 80% of WT levels, but there were also a 18.3% of the genes that gained H3K27me3 marks.
